# Intra-Articular Autologous Conditioned Serum Alone or Combined with Oral Cannabidiol in Dogs Undergoing Tibial Plateau Leveling Osteotomy: An Exploratory Randomized Controlled Trial

**DOI:** 10.3390/vetsci13101067

**Published:** 2026-10-10

**Authors:** Piyathida Ardaum, Narudee Kashemsant, Winutpuksinee Wibulchan, Julaporn Pooliam, Monchanok Vijarnsorn

**Affiliations:** 1 Kasetsart University Veterinary Teaching Hospital, Faculty of Veterinary Medicine, Kasetsart University, Bangkok 10900, Thailand; piyathida.ar@ku.th (P.A.); winutpuksinee.wi@ku.th (W.W.); 2 Department of Physiology, Faculty of Veterinary Medicine, Kasetsart University, Bangkok 10900, Thailand; narudee.k@ku.th; 3 Siriraj Research Data Management Unit, Research Department, Faculty of Medicine Siriraj Hospital, Mahidol University, Bangkok 10700, Thailand; julaporn.poo@mahidol.ac.th; 4 Department of Companion Animal Clinical Sciences, Faculty of Veterinary Medicine, Kasetsart University, Bangkok 10900, Thailand

**Keywords:** autologous conditioned serum, cannabidiol, tibial plateau leveling osteotomy, C-reactive protein, cranial cruciate ligament rupture, canine osteoarthritis, postoperative inflammation

## Abstract

Autologous conditioned serum (ACS) contains anti-inflammatory mediators, whereas cannabidiol (CBD) may have analgesic and immunomodulatory effects. This exploratory randomized trial evaluated intra-articular ACS, alone or combined with oral CBD, after tibial plateau leveling osteotomy in dogs. Twenty-seven dogs were randomized to placebo, ACS, or ACS + CBD (*n* = 9/group). Outcomes included C-reactive protein (CRP), peak vertical force, orthopedic assessment scores (lameness, joint mobility, pain on palpation, weight-bearing, and overall clinical condition), Canine Brief Pain Inventory, radiographic outcomes, and safety. CRP peaked on Day 1. In the linear mixed model using a heterogeneous first-order autoregressive covariance structure, treatment-group (*p* = 0.001) and visit (*p* < 0.001) effects were significant, whereas the treatment-group × visit interaction did not reach significance (*p* = 0.053). Exploratory comparisons showed lower CRP in ACS + CBD than placebo on Days 3 (adjusted *p* = 0.007) and 5 (adjusted *p* = 0.001), with model-dependent sensitivity findings. No consistent treatment-group differences were identified in clinical or structural outcomes. One ACS-treated dog developed a surgical site infection, and alkaline phosphatase activity was higher in ACS + CBD on Day 28. The CRP findings remain hypothesis-generating, with uncertain clinical relevance.

## Simple Summary

Cranial cruciate ligament rupture is a common cause of hindlimb lameness in dogs and is often treated with tibial plateau leveling osteotomy. This study explored whether intra-articular autologous conditioned serum, a blood-derived product containing anti-inflammatory substances, alone or combined with oral cannabidiol, was associated with differences in early recovery after surgery. Twenty-seven dogs were randomly assigned to placebo, autologous conditioned serum, or autologous conditioned serum plus cannabidiol and were assessed for 28 days. C-reactive protein, a blood marker of inflammation, was lower in the combined-treatment group than in the placebo group on postoperative Days 3 and 5 in the main analysis. Sensitivity analyses showed some variation depending on the statistical model used, particularly for the overall interaction and the Day 5 comparison. Other clinical and structural outcomes showed no consistent treatment-specific differences. Initial differences in joint mobility were not retained after accounting for baseline scores or in a nonparametric analysis. Short-term safety findings were generally similar among groups, although alkaline phosphatase activity was higher in the combined-treatment group on Day 28. Larger studies with longer follow-up are needed to determine whether these treatments provide clinically meaningful benefits after surgery.

## 1. Introduction

Cranial cruciate ligament rupture (CCLR) is one of the most common orthopedic conditions causing hindlimb lameness and progressive stifle osteoarthritis in dogs [1,2]. Tibial plateau leveling osteotomy (TPLO) is widely used to restore functional stability of the CCL-deficient stifle and is associated with progressive improvement in limb function after surgery [3,4]. Nevertheless, TPLO induces a measurable postoperative systemic inflammatory response, and radiographic osteoarthritis may continue to progress despite surgical stabilization of the joint [5,6]. Consequently, adjunctive treatments that may modulate postoperative inflammation while preserving functional recovery and osteotomy healing are of clinical interest in dogs undergoing TPLO.

Autologous conditioned serum (ACS) is an autologous blood-derived product prepared by incubating whole blood under conditions that increase concentrations of anti-inflammatory mediators, particularly interleukin-1 receptor antagonist (IL-1Ra), as well as selected cytokines and growth factors [7]. IL-1Ra competitively inhibits interleukin-1 signaling, a pathway involved in synovial inflammation and cartilage catabolism in osteoarthritis, providing a biological rationale for the intra-articular use of ACS [7]. In dogs, ACS has been shown to contain higher concentrations of IL-1Ra and selected growth factors than baseline plasma, although considerable interindividual variability in mediator concentrations has been reported [8]. In addition, ACS derived from dogs with naturally occurring osteoarthritis has demonstrated immunomodulatory and potentially regenerative cellular effects in vitro [9]. Collectively, these findings support further evaluation of ACS as an adjunctive biological treatment for joint inflammation in dogs; however, variability in its biological composition may contribute to heterogeneous treatment responses.

Cannabidiol (CBD) has also received increasing attention in veterinary medicine because of its potential analgesic and immunomodulatory properties. In dogs with naturally occurring osteoarthritis, oral CBD has been associated with improvements in pain and mobility-related outcomes in randomized clinical studies, including one study using a dose of 2 mg/kg every 12 h [10,11,12,13]. In addition to these clinical findings, CBD has been reported to influence inflammatory responses, and canine immune cells have been shown to express cannabinoid receptors CB1 and CB2, providing a biological basis for potential immunomodulatory effects in dogs [14]. Although ACS and CBD each have biological rationales that may be relevant to postoperative inflammation, whether their combined use provides additional biomarker or clinical benefit after TPLO has not been established [15].

Serum C-reactive protein (CRP) is a major positive acute-phase protein in dogs and increases rapidly following surgical trauma, making it a sensitive marker of the systemic inflammatory response during early postoperative recovery [5,16,17,18,19]. After TPLO, serum CRP typically rises markedly within the first 24 h and subsequently declines during uncomplicated recovery, supporting its use for longitudinal assessment of the systemic postoperative inflammatory response [5]. Although CRP is not specific to intra-articular inflammation or clinical function, its sensitivity to systemic inflammatory activity makes it a useful biomarker for monitoring early postoperative inflammatory changes [16,17]. Accordingly, this exploratory randomized, double-blinded, placebo-controlled clinical trial examined whether intra-articular ACS, administered alone or in combination with oral CBD, was associated with differences in early postoperative recovery across multiple outcome domains. These included serum CRP as a biomarker of systemic inflammation, limb loading, orthopedic clinical scores, owner-reported pain and function, radiographic osteoarthritis, osteotomy healing, and short-term safety, including adverse events and laboratory variables, during the first 28 postoperative days. We hypothesized that different treatments would result in distinct postoperative outcomes. Within an exploratory framework, we took a comprehensive approach by evaluating the longitudinal CRP response together with clinical, biomechanical, radiographic, and safety measures to characterize treatment patterns and generate hypotheses about their biological and clinical relevance.

## 2. Materials and Methods

### 2.1. Study Design, Exploratory Framework, and Ethical Approval

This prospective, block-randomized, double-blinded, placebo-controlled clinical trial was conducted at the Kasetsart University Veterinary Teaching Hospital (KUVTH), Bangkok, Thailand, between April 2022 and February 2024. Client-owned dogs diagnosed with unilateral cranial cruciate ligament rupture (CCLR) and scheduled to undergo tibial plateau leveling osteotomy (TPLO) were consecutively screened for eligibility. The trial evaluated intra-articular autologous conditioned serum (ACS), administered alone or in combination with oral cannabidiol (CBD), across multiple outcome domains during early postoperative recovery after TPLO. Prospectively collected measures included inflammatory biomarkers, biomechanical and clinical assessments, owner-reported outcomes, radiographic osteoarthritis and osteotomy-healing assessments, and laboratory safety variables during the first 28 postoperative days. Because the original protocol did not prespecify a single primary outcome or a primary-secondary outcome hierarchy, the trial is reported as an exploratory multi-outcome randomized clinical trial.

Twenty-seven dogs were enrolled and allocated equally among the three treatment groups. Consistent with the exploratory multi-outcome design described above, analyses were interpreted as hypothesis-generating rather than as confirmatory tests of efficacy for any single outcome. The sample size also limited the precision of treatment-effect estimates and the ability to detect modest between-group differences.

The study protocol was approved by the Institutional Animal Care and Use Committee (IACUC) of the Faculty of Veterinary Medicine, Kasetsart University (approval no. ACKU65-VET-029; approved 7 April 2022). Written informed owner consent was obtained before enrollment.

### 2.2. Study Population, Withdrawal Criteria, and Post-Randomization Events

Dogs were eligible for inclusion if they had unilateral CCLR confirmed by orthopedic examination (cranial drawer and/or tibial compression test) together with radiographic findings, were skeletally mature, weighed ≥20 kg, and had a body condition score (BCS) of 4/9 to 8/9. Dogs with a history of contralateral TPLO were eligible only if complete radiographic healing and absence of clinically detectable contralateral lameness were documented at enrollment.

Exclusion criteria applied before randomization included neurologic deficits; clinically relevant systemic disease, including hepatic, renal, or endocrine disorders; American Society of Anesthesiologists (ASA) physical status >3; active infection; clinically relevant metabolic abnormalities; or excessive anesthetic risk as determined by the attending anesthesiologist.

Prospectively defined withdrawal criteria applied after randomization included implant failure requiring revision surgery, surgical site infection, fracture, persistent uncontrolled pain, organ dysfunction, septicemia, or documented owner non-compliance. These events were considered capable of independently affecting inflammatory, clinical, biomechanical, or radiographic outcomes and therefore precluded continued participation in the planned outcome assessments. Dogs meeting withdrawal criteria remained in the safety population. Their inclusion in analyses of study outcomes was handled according to the analysis-population procedures described in Section 2.9.1.

### 2.3. Randomization and Blinding

Dogs were randomly assigned to one of three treatment groups: (1) intra-articular saline plus oral placebo (placebo group); (2) intra-articular ACS plus oral placebo (ACS group); or (3) intra-articular ACS combined with oral CBD (ACS + CBD group).

Dogs were stratified according to body condition status (obese vs. non-obese) before randomization, with obesity defined as BCS ≥ 7/9. Within each stratum, treatment allocation was generated by computer using permuted blocks of three. Allocation concealment was maintained using sequentially numbered, opaque, sealed envelopes opened only after enrollment and immediately before preparation of the assigned intervention. The allocation sequence was generated and retained by an independent investigator who did not participate in clinical assessment, treatment administration, or outcome analysis.

Owners, surgeons, clinical evaluators performing orthopedic examinations and force-plate assessments, the radiographic evaluator, laboratory personnel, and statistical analysts were blinded to treatment allocation. Intra-articular syringes containing ACS or saline were externally masked to conceal their contents. Treatment syringes and oral capsules were prepared by an independent investigator who did not participate in surgery, postoperative care, outcome assessment, or data analysis. CBD capsules (99.8% purified isolate; Δ9-tetrahydrocannabinol not detected by high-performance liquid chromatography) and placebo capsules were identical in appearance.

### 2.4. Perioperative Management and Concomitant Care

All dogs received standardized anesthetic, analgesic, antimicrobial, and rehabilitation protocols irrespective of treatment allocation. Lactated Ringer’s solution (ANB Laboratories Co., Ltd., Bangkok, Thailand) was administered intravenously at 10 mL/kg/h during anesthesia. Premedication consisted of morphine (0.5 mg/kg IM; M&H Manufacturing Co., Ltd., Samut Prakan, Thailand) and midazolam (0.2 mg/kg IM; Siam Bioscience Co., Ltd., Bang Yai, Nonthaburi, Thailand), and amoxicillin–clavulanic acid (20 mg/kg IV; Siam Bheasach Co., Ltd., Bangkok, Thailand) was administered for antimicrobial prophylaxis. Anesthesia was induced with propofol (2–4 mg/kg IV; Troikaa Pharmaceuticals Ltd., Dehradun, India) and maintained with isoflurane (Piramal Critical Care, Inc., Bethlehem, PA, USA) in oxygen.

Before surgical incision, epidural bupivacaine (1 mg/kg; 0.5% [5 mg/mL]; AstraZeneca AB, Södertälje, Sweden) was administered. Intraoperative analgesia consisted of a morphine–lidocaine–ketamine constant-rate infusion (morphine, 0.3 mg/kg/h; lidocaine, 3 mg/kg/h; ketamine, 0.6 mg/kg/h) preceded by loading doses of lidocaine (1 mg/kg IV; L.B.S. Laboratory Co., Ltd., Lam Luk Ka, Pathum Thani, Thailand) and ketamine (1 mg/kg IV; Siegfried Hameln GmbH, Hameln, Germany). Postoperatively, dogs received carprofen (2.2 mg/kg SC; Zoetis Manufacturing & Research Spain, S.L., Vall de Bianya, Girona, Spain), followed by carprofen (2.2 mg/kg PO every 12 h; Zoetis Inc., Lincoln, NE, USA) for 3 days, and gabapentin (10 mg/kg PO every 12 h; Berlin Pharmaceutical Industry Co., Ltd., Bangkok, Thailand) for 7 days.

Postoperative pain was assessed using a modified version of the Colorado State University Canine Acute Pain Scale (used with permission). Rescue analgesia was administered when the pain score exceeded 2 and consisted of ketamine (1 mg/kg IV) and/or morphine (0.3 mg/kg IM), as clinically indicated. All rescue analgesic administration was recorded as concomitant treatment.

Postoperative rehabilitation was standardized across groups and consisted of cold-compression therapy beginning on Day 2, interferential-current therapy and laser therapy from Days 3 to 7, followed by weekly laser-therapy sessions for 4 weeks. Procedures were delivered according to a written institutional protocol by personnel blinded to treatment allocation.

### 2.5. Surgical Procedure

TPLO was performed by experienced surgeons using a standard TPLO technique. Preoperative radiographs were obtained to determine the tibial plateau angle (TPA) and plan osteotomy positioning. A medial parapatellar arthrotomy was performed, ruptured cranial cruciate ligament fibers were excised, and partial meniscectomy was performed when a meniscal tear was identified. A curved radial osteotomy was created and rotated to achieve a postoperative TPA of approximately 5°. Fixation was achieved using a locking TPLO plate (TPLO Clover plate, Fixin^®^, Intrauma S.p.A., Bruino, Italy).

Immediately before skin closure on Day 0, the assigned intra-articular treatment was administered aseptically into the operated stifle joint: ACS (2 mL) in the ACS and ACS + CBD groups or saline (2 mL) in the placebo group.

### 2.6. Preparation of Autologous Conditioned Serum

ACS was prepared using the Orthokine^®^ vet IRAP II 10-ACS system (Orthogen Veterinary GmbH, Düsseldorf, Germany) according to the manufacturer’s instructions. Twenty milliliters of whole blood were collected aseptically from each dog and incubated at 37 °C for 7 h in a dual-chamber syringe containing medical-grade glass beads designed to promote the production of anti-inflammatory mediators, including interleukin-1 receptor antagonist (IL-1Ra). Samples were centrifuged at 3000× *g* for 10 min. The conditioned serum was harvested aseptically, divided into aliquots, and stored at −20 °C until administration. ACS was thawed immediately before use and was not refrozen. For each dog, aliquots administered on Days 0 and 14 were derived from the same preparation for each dog.

Concentrations of IL-1Ra and other cytokines were not measured in individual ACS preparations; therefore, ACS administration was standardized by injection volume rather than by mediator concentration.

### 2.7. Treatment Protocol and Dose Rationale

Dogs in the placebo group received intra-articular saline (2 mL) on postoperative Days 0 and 14 together with oral placebo capsules for 28 days. Dogs in the ACS group received intra-articular ACS (2 mL) on Days 0 and 14 together with oral placebo capsules for 28 days. Dogs in the ACS + CBD group received intra-articular ACS (2 mL) on Days 0 and 14 together with oral CBD at 2 mg/kg every 12 h for 28 days. The oral CBD dose was selected based on a previous pharmacokinetic and randomized clinical study in dogs with naturally occurring osteoarthritis, in which a dosing regimen of 2 mg/kg every 12 h was evaluated for tolerability and potential effects on pain and activity [10]. Oral administration began on the evening of surgery and continued for 28 consecutive days. Treatment compliance was assessed using capsule counts and owner diaries, and missed doses or protocol deviations were recorded.

A fixed intra-articular volume of 2 mL was administered to all dogs irrespective of body weight. This volume was selected based on its clinical feasibility for injection into the canine stifle and previous canine research using a 2 mL intra-articular injection volume in this joint [20]. The injection volume was standardized across treatment groups to minimize procedural variability.

All intra-articular injections were performed aseptically using a 22-gauge needle through a medial approach. Intra-articular needle placement was confirmed by aspiration of synovial fluid before slow administration of the assigned treatment. Day 14 injections were performed by the attending veterinarian under manual restraint without sedation. Personnel involved in outcome assessment remained blinded to treatment allocation.

### 2.8. Outcome Measures

#### 2.8.1. Exploratory Biomarker Outcome

##### Serum C-Reactive Protein (CRP)

Serum CRP concentration was evaluated longitudinally as an exploratory biomarker of the acute postoperative systemic inflammatory response and to explore potential treatment-associated differences during early recovery. Measurements were obtained preoperatively (Day 0) and on postoperative Days 1, 3, 5, 7, 14, and 28. Serum CRP concentrations were analyzed in batches using the Catalyst^®^ CRP Test (IDEXX Laboratories, Inc., Westbrook, ME, USA), a validated sandwich immunoassay based on gold-conjugate technology. The reference interval was 0–1 mg/dL.

#### 2.8.2. Exploratory Clinical, Functional, and Radiographic Outcomes

##### Force Plate Gait Analysis

Force-plate assessments were performed on Days 0, 7, 14, and 28 using a 10 m walkway with two strain-gauge force plates embedded side by side (Model OR6-6; Advanced Mechanical Technology, Watertown, MA, USA). Dogs underwent a 5 min acclimation period. Each dog was guided at a trot by a handler within the prespecified velocity range of 1.2–2.2 m/s; passes outside this range or showing visible acceleration, deceleration, targeting, distraction, overlapping paw contact, or incomplete contact of the operated pelvic limb were rejected. Six valid passes were obtained per session. Signals were processed using Cortex 4.0 software (Motion Analysis Corporation, Santa Rosa, CA, USA). Peak vertical force (PVF) of the operated pelvic limb was averaged across the six valid passes and normalized to body weight (%BW). For longitudinal analysis, change from baseline (ΔPVF; follow-up value minus Day 0 value) was calculated for postoperative Days 7, 14, and 28, with positive values indicating increased loading of the operated limb relative to baseline. All measurements were obtained by a blinded examiner.

##### Orthopedic Assessment Score (OAS)

Clinical lameness and stifle-related function were assessed on Days 0, 1, 3, 5, 7, 14, and 28 using a five-domain orthopedic scoring system comprising lameness, joint mobility, pain on palpation, weight-bearing, and overall clinical condition [21] (Table 1). Each domain was graded on a 5-point ordinal scale, with higher scores indicating greater clinical impairment. The same blinded clinician performed all assessments to minimize interobserver variability. For longitudinal analysis, change from baseline was calculated for each domain as the follow-up score minus the Day 0 score, with negative values indicating improvement relative to baseline. Because the scores were ordinal, analyses that treated these scales as approximately interval were interpreted cautiously.

##### Canine Brief Pain Inventory (CBPI)

Owner-reported pain severity and pain-related interference with function were assessed using the Canine Brief Pain Inventory (CBPI) at baseline (Day 0) and on postoperative Days 14 and 28. The CBPI comprised the Pain Severity Score (PSS), Pain Interference Score (PIS), and a global Quality-of-Life (QOL) assessment. Higher PSS and PIS values indicated greater pain severity and interference with function, whereas higher QOL scores indicated better perceived quality of life. For longitudinal analysis, changes from baseline were calculated for each outcome, with negative changes in PSS and PIS and positive changes in QOL indicating improvement relative to baseline.

##### Radiographic Osteoarthritis Assessment

Standard mediolateral and craniocaudal radiographs of the operated stifle were obtained at baseline (Day 0) and postoperative Day 28. Radiographs were anonymized and evaluated in randomized order by a blinded radiographic assessor. Osteoarthritic changes were assessed using a subjective radiographic scoring system adapted from Innes et al. [22], with ordinal scores assigned for joint effusion, osteophytosis, intra-articular mineralization, and tibial subchondral sclerosis (Supplementary Table S1). In addition, overall OA severity was classified using a previously described 0–5 semiquantitative scoring system based on the presence and distribution of osteophytes and subchondral bone changes [23] (Supplementary Table S2). Higher scores indicated greater radiographic osteoarthritic severity.

##### Osteotomy-Healing Assessment

Immediate postoperative and Day 28 mediolateral and craniocaudal radiographs were anonymized and assessed in randomized order by the blinded radiographic evaluator. Healing was evaluated using a 10-point TPLO scoring system assessing cortical continuity, osteotomy-line visibility, callus formation and remodeling, and rounding of the distal osteotomy margin, together with a 5-point ordinal scale estimating the extent of radiographic bridging [22,24,25] (Supplementary Tables S3 and S4). Higher scores indicated more advanced healing. Between-group comparisons at Day 28 were used to explore potential treatment-associated differences in radiographic healing, whereas within-group changes from the immediate postoperative assessment were used descriptively to characterize expected healing progression over time.

#### 2.8.3. Safety Outcomes

All randomized dogs that received at least one dose of the assigned study intervention were included in the safety population and followed through postoperative Day 28. Adverse events, postoperative complications, rescue treatments, treatment discontinuations, and clinically relevant protocol deviations were recorded throughout the study. Hematology and serum biochemistry variables, including packed cell volume (PCV), white blood cell count (WBC), platelet count, alkaline phosphatase (ALP), alanine aminotransferase (ALT), blood urea nitrogen (BUN), creatinine, total protein, and albumin, were evaluated on Days 0, 3, 14, and 28. All available safety observations were retained regardless of subsequent withdrawal from planned study-outcome assessments.

### 2.9. Statistical Analysis

#### 2.9.1. Analysis Populations and Baseline Analyses

All statistical analyses were performed using IBM SPSS Statistics version 31 (IBM Corp., Armonk, NY, USA). The safety population included all randomized dogs that received at least one assigned intervention. The exploratory CRP analysis used a modified intention-to-treat (mITT) approach, including all randomized dogs with available CRP measurements and retaining dogs according to their randomized treatment allocation. Analyses of clinical, biomechanical, owner-reported, radiographic, and osteotomy-healing outcomes used available measurements from dogs who remained in the planned study-outcome follow-up.

One dog in the ACS group developed a postoperative surgical site infection that was clinically detected on Day 3 and met a prospectively defined withdrawal criterion. The dog was withdrawn from subsequent planned outcome assessments but remained in the safety population. CRP measurements obtained before detection of the infection (Days 0 and 1) were retained in the exploratory CRP analysis. No further CRP measurements were collected from Day 3 onward following withdrawal; therefore, no post-withdrawal CRP observations were available for inclusion in the longitudinal analysis. No imputation was performed. All available adverse-event and laboratory safety observations for this dog were retained in the safety analyses.

Baseline continuous variables were summarized as mean ± standard deviation, and categorical variables were summarized as counts and percentages. Any statistical comparisons of baseline characteristics among randomized groups were interpreted descriptively and were not used to establish baseline equivalence or to determine inclusion of variables in subsequent models.

Longitudinal continuous outcomes were analyzed using repeated-measures linear mixed models estimated by restricted maximum likelihood. Treatment group, categorical visit, and the treatment-group × visit interaction were included as fixed effects, with visit specified as a repeated measure within dog. Alternative within-dog covariance structures were evaluated for each outcome, and the structure yielding the lowest Akaike information criterion (AIC) among successfully fitted candidate models was selected for the final model. Denominator degrees of freedom were estimated using the Satterthwaite approximation. Estimated marginal means (EMMs) with 95% confidence intervals (CIs) were obtained for each treatment group at each visit. Pairwise comparisons between treatment groups within visits and between visits within treatment groups were adjusted using the Bonferroni method. These visit-specific comparisons were interpreted exploratorily, particularly when the overall treatment-group × visit interaction was not statistically significant. Mixed models incorporated all available observations within the relevant analysis population without single-value imputation.

#### 2.9.2. Exploratory CRP Analysis

Serum CRP concentrations measured on Days 0, 1, 3, 5, 7, 14, and 28 were analyzed longitudinally in the modified intention-to-treat (mITT) population using the repeated-measures linear mixed-model framework described above. Candidate within-dog covariance structures included unstructured (UN), first-order autoregressive [AR(1)], compound symmetry (CS), heterogeneous first-order autoregressive [ARH(1)], and heterogeneous compound symmetry (CSH) structures. Among these models, ARH(1) yielded the lowest Akaike information criterion (AIC) and was selected as the main covariance structure for CRP analysis.

Sensitivity analyses using alternative covariance structures were performed to assess the robustness of the findings. Additional post hoc sensitivity analyses were conducted using natural-log-transformed CRP [ln(CRP)] with UN, ARH(1), and CSH structures. The same fixed-effects specification, estimation method, degrees-of-freedom approximation, and multiplicity adjustment were retained across analyses. All fitted covariance models converged without warnings. Detailed sensitivity-analysis results are provided in Supplementary Table S6.

#### 2.9.3. Exploratory Clinical, Functional, and Radiographic Analyses

##### Peak Vertical Force

Change from baseline in PVF (ΔPVF; follow-up value minus Day 0 value) at Days 7, 14, and 28 was analyzed using the general mixed-model framework, with an unstructured within-dog covariance structure selected based on the lowest AIC among successfully fitted candidate structures. Positive ΔPVF values indicated increased loading of the operated limb relative to baseline.

##### Orthopedic Assessment Score (OAS)

Changes from baseline in individual OAS domains were analyzed separately using the general mixed-model framework. A first-order autoregressive [AR(1)] within-dog covariance structure was selected for each domain based on the lowest AIC among successfully fitted candidate structures. Negative changes indicated improvement. Because the OAS domains were ordinal, analyses treating these scales as approximately interval were interpreted cautiously and considered exploratory.

Because baseline joint-mobility scores were numerically higher in the ACS + CBD group, indicating greater baseline impairment, a post hoc baseline-adjusted sensitivity analysis was performed for this domain using postoperative joint-mobility scores as the outcome and baseline joint-mobility score as a covariate, with treatment group, visit, and the treatment-group × visit interaction included as fixed effects. An AR(1) within-dog covariance structure was used. Given the ordinal nature of the joint-mobility score, a nonparametric sensitivity analysis of change-from-baseline scores was also performed using the Kruskal–Wallis test to compare treatment groups at each postoperative visit.

##### Canine Brief Pain Inventory (CBPI)

Changes from baseline in PSS, PIS, and QOL at Days 14 and 28 were analyzed separately using the general mixed-model framework, with an unstructured within-dog covariance structure selected for each outcome based on the lowest AIC among successfully fitted candidate structures. Negative changes in PSS and PIS and positive changes in QOL indicated improvement relative to baseline. Because the global QOL assessment was ordinal, analysis treating this scale as approximately interval was interpreted cautiously.

##### Radiographic Osteoarthritis

Radiographic osteoarthritis outcomes were analyzed nonparametrically. Between-group comparisons at Days 0 and 28 used the Kruskal–Wallis test, with Bonferroni-adjusted pairwise comparisons where applicable. Within-group changes from Day 0 to Day 28 were evaluated using the Wilcoxon signed-rank test. Ordinal outcomes were summarized as median (range), and within-group changes were interpreted descriptively as changes over time rather than as evidence of comparative treatment effects.

##### Osteotomy-Healing Assessment

Osteotomy-healing outcomes were summarized as median (range). Between-group comparisons at Day 28 used the Kruskal–Wallis test, with Bonferroni-adjusted pairwise comparisons where applicable. Within-group changes from the immediate postoperative assessment to Day 28 were evaluated using the Wilcoxon signed-rank test and were interpreted descriptively as healing progression over time.

#### 2.9.4. Laboratory Safety Analyses

PCV, WBC, platelet count, BUN, creatinine, total protein, and albumin measured on Days 0, 3, 14, and 28 were analyzed using the general mixed-model framework, with an unstructured within-dog covariance structure selected for each variable based on the lowest AIC among successfully fitted candidate structures.

ALT and ALP activities were analyzed nonparametrically because their distributions were not considered appropriate for parametric modeling. Between-group comparisons at each visit used the Kruskal–Wallis test, with Bonferroni-adjusted pairwise comparisons where applicable. Within-group comparisons across visits were performed using the Friedman test, with Bonferroni-adjusted pairwise comparisons where applicable.

#### 2.9.5. Multiplicity and Reporting

No single multiplicity adjustment was applied across the distinct outcome families evaluated in this exploratory multi-outcome trial. Visit-specific and outcome-specific comparisons were considered exploratory, and interpretation considered treatment-effect estimates and 95% CIs, where applicable, alongside *p*-values. Where multiple pairwise comparisons were performed within an outcome and visit, *p*-values were adjusted using the Bonferroni method as specified above. All statistical tests were two-sided, with *p* < 0.05 used as the threshold for statistical significance within individual analyses; adjusted *p*-values are reported where multiplicity correction was applied. Findings reaching statistical significance within individual exploratory analyses were not interpreted as confirmatory evidence of treatment efficacy.

## 3. Results

### 3.1. Enrollment and Baseline Characteristics

**Figure 1:**
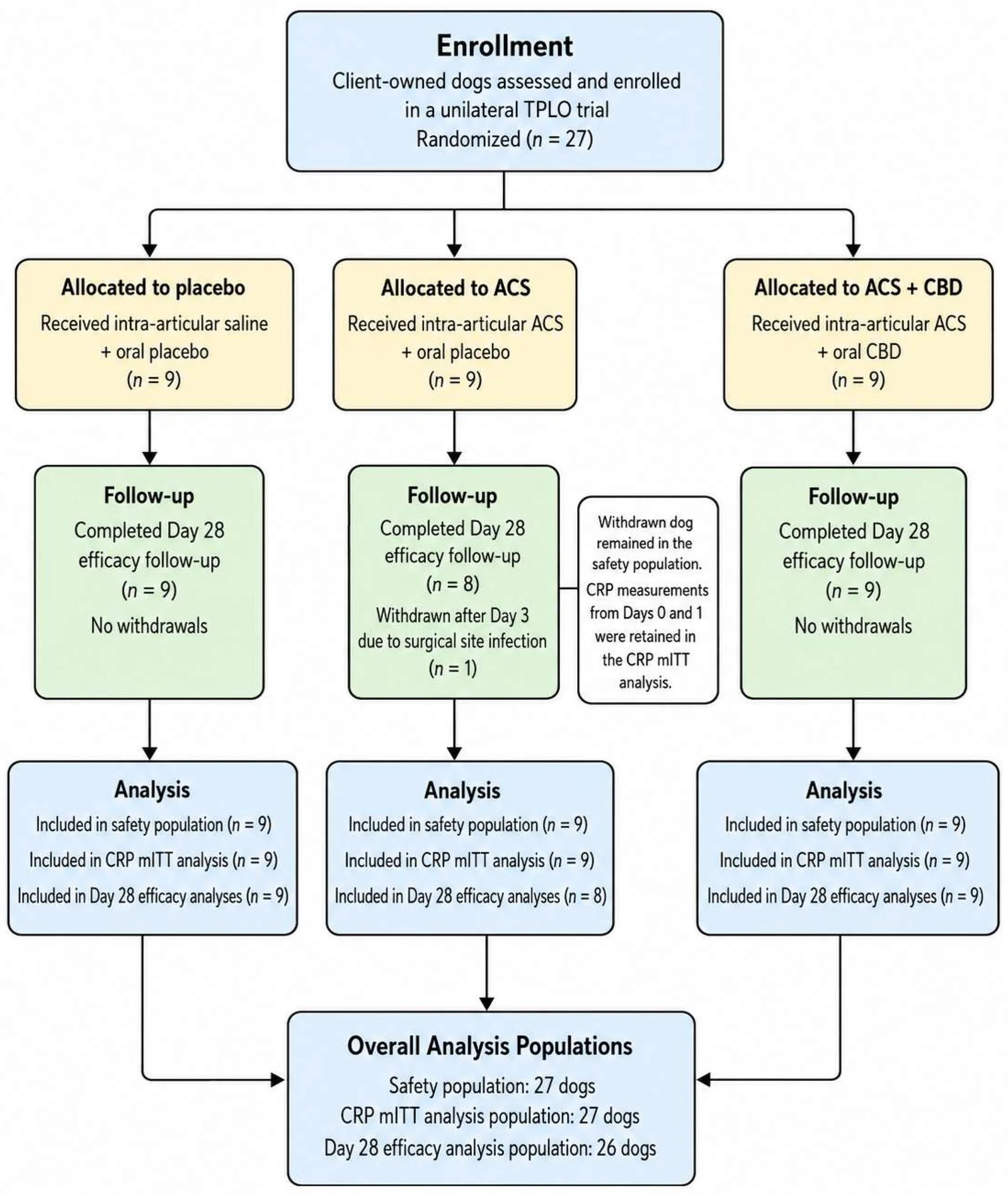
Flow diagram of enrollment, allocation, follow-up, and analysis populations. Twenty-seven dogs were randomized equally into placebo, ACS, or ACS + CBD groups (*n* = 9/group). One dog in the ACS group was withdrawn from efficacy assessments after developing a surgical site infection on Day 3 but remained in the safety population. This dog contributed CRP data for Days 0 and 1, yielding a CRP mITT population of 27 dogs; 26 dogs completed the Day 28 follow-up and were included in the final efficacy analysis.

A total of 27 client-owned dogs scheduled to undergo unilateral tibial plateau leveling osteotomy (TPLO) were enrolled and randomly assigned in equal proportions to the placebo, ACS, or ACS + CBD groups (*n* = 9 per group) (Figure 1). One dog assigned to the ACS group developed a postoperative surgical site infection detected on Day 3 and was withdrawn from subsequent planned outcome assessments according to the predefined withdrawal criteria. All 27 randomized dogs were retained in the safety population. For the modified intention-to-treat CRP analysis, all 27 dogs contributed data; the withdrawn dog contributed CRP measurements obtained before clinical detection of the infection on Days 0 and 1. Twenty-six dogs completed follow-up through Day 28 and contributed to the clinical, biomechanical, owner-reported, radiographic, and osteotomy-healing analyses: placebo, *n* = 9; ACS, *n* = 8; and ACS + CBD, *n* = 9.

Baseline demographic and clinical characteristics are summarized in Table 2. Baseline comparisons were descriptive, and no statistically significant differences among randomized groups were identified for the variables presented in Table 2; baseline radiographic osteoarthritis variables are reported separately. Baseline serum CRP concentrations showed no statistically significant between-group difference and were within the reference interval (*p* = 0.970), and baseline peak vertical force of the operated pelvic limb also showed no statistically significant between-group difference (*p* = 0.584).

No statistically significant between-group differences were identified in baseline Orthopedic Assessment Score domains. Joint-mobility scores were numerically higher in the ACS + CBD group, indicating greater baseline impairment, although the between-group comparison did not reach statistical significance (*p* = 0.055). This baseline imbalance was considered when interpreting subsequent exploratory findings for the joint-mobility domain. No statistically significant baseline differences were detected for the Canine Brief Pain Inventory Pain Severity Score, Pain Interference Score, or Quality-of-Life Score (all *p* > 0.05).

### 3.2. Serum C-Reactive Protein

**Figure 2:**
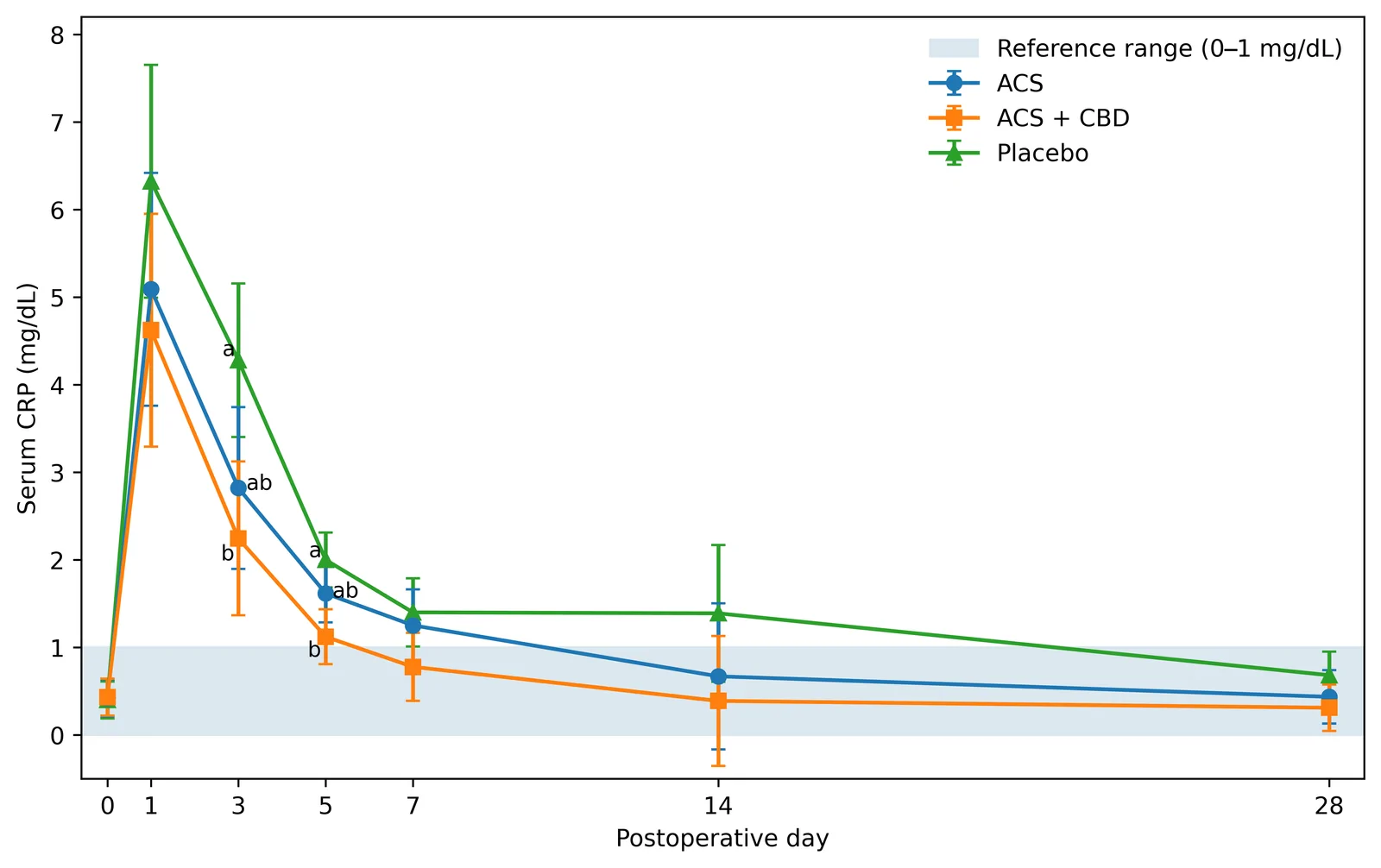
Estimated marginal mean serum C-reactive protein (CRP) concentrations from baseline (Day 0) through postoperative Day 28 in dogs undergoing tibial plateau leveling osteotomy and assigned to placebo, autologous conditioned serum (ACS), or ACS plus cannabidiol (ACS + CBD). Error bars represent 95% confidence intervals derived from the repeated-measures linear mixed model using a heterogeneous first-order autoregressive [ARH(1)] within-dog covariance structure. The shaded area indicates the laboratory reference interval (0–1 mg/dL). Pairwise treatment-group comparisons within each visit were adjusted using the Bonferroni method and were interpreted as exploratory because the overall treatment-group × visit interaction was not statistically significant (*p* = 0.053). Within a visit, treatment groups that do not share a letter differ significantly in Bonferroni-adjusted pairwise comparisons (adjusted *p* < 0.05). In exploratory pairwise comparisons, CRP concentrations were lower in the ACS + CBD group than in the placebo group on Day 3 (adjusted *p* = 0.007) and Day 5 (adjusted *p* = 0.001).

Serum CRP concentrations increased after surgery in all treatment groups, reached their highest estimated marginal values on postoperative Day 1, and subsequently declined toward baseline levels over the 28-day follow-up period (Figure 2, Table 3). Baseline CRP concentrations were similar among groups and were within the reference interval.

In the longitudinal repeated-measures linear mixed model using the ARH(1) covariance structure, significant effects of treatment group (F = 7.984, *p* = 0.001) and visit (F = 54.582, *p* < 0.001) were observed, whereas the treatment-group × visit interaction did not reach statistical significance (F = 1.944, *p* = 0.053). Given the non-significant interaction, treatment-group comparisons within individual visits were interpreted as exploratory.

On postoperative Day 1, estimated marginal mean CRP concentrations were 6.322 mg/dL in the placebo group, 5.089 mg/dL in the ACS group, and 4.622 mg/dL in the ACS + CBD group. Although CRP concentrations were numerically lower in both ACS-treated groups than in the placebo group, neither comparison was statistically significant after Bonferroni adjustment (ACS vs. placebo: mean difference, −1.233 mg/dL; 95% CI, −3.574 to 1.107; adjusted *p* = 0.569; ACS + CBD vs. placebo: mean difference, −1.700 mg/dL; 95% CI, −4.040 to 0.640; adjusted *p* = 0.224). No difference was detected between the ACS and ACS + CBD groups (adjusted *p* = 1.000).

In exploratory visit-specific comparisons, CRP concentrations were lower in the ACS + CBD group than in the placebo group on postoperative Day 3 (mean difference, −2.033 mg/dL; 95% CI, −3.574 to −0.493; adjusted *p* = 0.007) and Day 5 (mean difference, −0.878 mg/dL; 95% CI, −1.427 to −0.328; adjusted *p* = 0.001). The ACS group did not differ significantly from the placebo group at either Day 3 (adjusted *p* = 0.079) or Day 5 (adjusted *p* = 0.289). No statistically significant between-group differences were detected at the remaining postoperative visits. By Day 28, CRP concentrations had declined toward baseline levels in all three groups.

Sensitivity analyses using alternative covariance structures and natural-log-transformed CRP showed some variation in inferential results according to model specification. Detailed results are provided in Supplementary Table S6.

### 3.3. Clinical, Functional, and Radiographic Outcomes

#### 3.3.1. Peak Vertical Force

**Figure 3:**
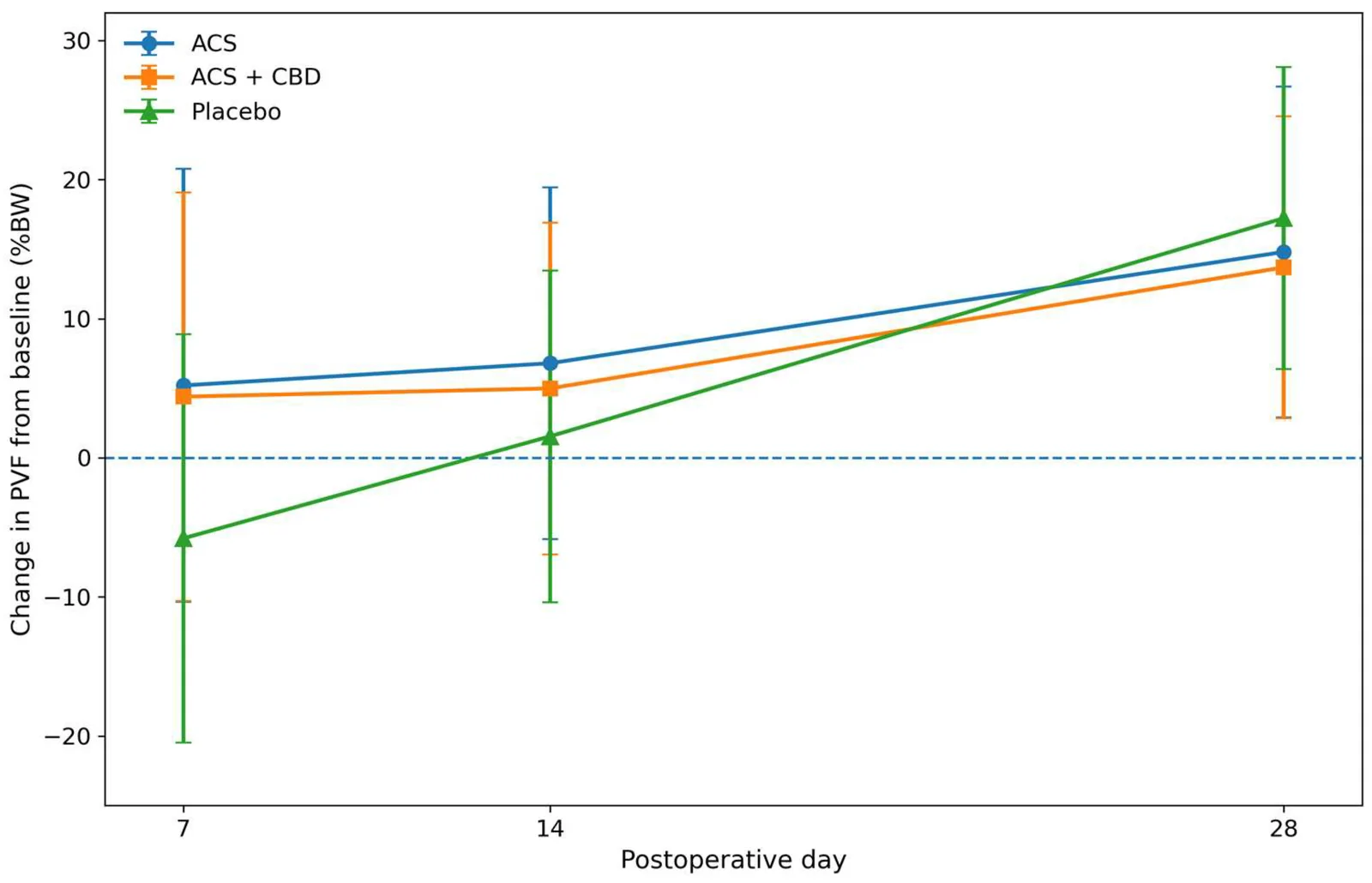
Estimated marginal mean change from baseline in peak vertical force (ΔPVF) of the operated pelvic limb from postoperative Days 7 to 28 in dogs undergoing tibial plateau leveling osteotomy and assigned to placebo, autologous conditioned serum (ACS), or ACS plus cannabidiol (ACS + CBD). Values are expressed as a percentage of body weight (%BW), and error bars represent 95% confidence intervals derived from the final repeated-measures linear mixed model using an unstructured within-dog covariance structure. The horizontal dashed line represents no change from baseline (ΔPVF = 0); positive values indicate increased loading of the operated limb relative to baseline. No statistically significant between-group differences were detected at any postoperative visit after Bonferroni adjustment.

Change from baseline in peak vertical force (ΔPVF) of the operated pelvic limb increased over the postoperative period, consistent with progressively increased limb loading relative to baseline (Figure 3, Table 4). In the repeated-measures linear mixed model, there was a significant effect of visit (F = 21.866, *p* < 0.001), whereas neither the treatment-group effect (F = 0.174, *p* = 0.842) nor the treatment-group × visit interaction (F = 1.854, *p* = 0.151) was statistically significant. Estimated marginal mean ΔPVF values at Day 28 were 17.228%BW in the placebo group, 14.798%BW in the ACS group, and 13.689%BW in the ACS + CBD group. No statistically significant between-group differences were identified at Days 7, 14, or 28 after Bonferroni adjustment. Overall, operated-limb loading increased over time, with no statistically significant treatment-group differences detected during the 28-day postoperative period.

#### 3.3.2. Orthopedic Assessment Scores

Changes from baseline across the Orthopedic Assessment Score domains generally indicated improvement over the postoperative follow-up period. Significant visit effects were detected for lameness (F = 13.256, *p* < 0.001), joint mobility (F = 4.508, *p* = 0.001), pain on palpation (F = 2.596, *p* = 0.029), weight-bearing (F = 9.650, *p* < 0.001), and overall clinical condition (F = 7.330, *p* < 0.001). Treatment-group × visit interactions were not statistically significant for any OAS domain (all *p* > 0.05). Treatment-group main effects were also not statistically significant for lameness (*p* = 0.358), pain on palpation (*p* = 0.804), weight-bearing (*p* = 0.186), or overall clinical condition (*p* = 0.690).

Joint mobility was the only OAS domain with a statistically significant main effect of treatment group in the original change-from-baseline analysis (F = 3.840, *p* = 0.033), although the treatment-group × visit interaction was not statistically significant (F = 1.457, *p* = 0.167) (Table 5). Given the non-significant interaction, treatment-group comparisons within individual visits were interpreted exploratorily. In Bonferroni-adjusted visit-specific comparisons, the ACS + CBD group showed a larger decrease in joint-mobility score from baseline than the placebo group on Day 7 (mean difference, −1.667 points; adjusted *p* = 0.022) and Day 28 (mean difference, −1.667 points; adjusted *p* = 0.022). At Day 28, the ACS + CBD group also showed a larger decrease from baseline than the ACS group (mean difference, −1.641 points; adjusted *p* = 0.042).

Because baseline joint-mobility scores were numerically higher in the ACS + CBD group, indicating greater baseline impairment, a post hoc baseline-adjusted sensitivity analysis was performed. In this analysis, baseline joint-mobility score was significantly associated with postoperative joint-mobility scores (F = 4.341, *p* = 0.044), whereas neither the main effect of treatment group (F = 0.191, *p* = 0.827) nor the treatment-group × visit interaction (F = 1.485, *p* = 0.156) was statistically significant. No statistically significant between-group differences were detected at any postoperative visit, including Days 7 and 28 (adjusted *p* = 0.176 and *p* = 0.189, respectively).

A nonparametric sensitivity analysis of change-from-baseline joint-mobility scores, performed because of the ordinal nature of the scale, similarly identified no statistically significant between-group differences at any postoperative visit (Kruskal–Wallis tests, all *p* > 0.05). Thus, the visit-specific differences observed in the original change-from-baseline analysis were not retained after adjustment for baseline joint mobility or in the nonparametric sensitivity analysis.

#### 3.3.3. Canine Brief Pain Inventory (CBPI)

Owner-reported CBPI outcomes showed improvement over the postoperative follow-up period. For change from baseline in the Pain Severity Score (PSS), there was a statistically significant effect of visit (F = 9.542, *p* = 0.005), whereas neither the main effect of treatment group (F = 1.443, *p* = 0.256) nor the treatment-group × visit interaction (F = 0.112, *p* = 0.894) was statistically significant. Similarly, change in the Pain Interference Score (PIS) showed a statistically significant visit effect (F = 12.471, *p* = 0.002), with no statistically significant main effect of treatment group (F = 1.732, *p* = 0.199) or treatment-group × visit interaction (F = 0.582, *p* = 0.567). Change in Quality-of-Life (QOL) scores also showed a statistically significant effect of visit (F = 28.367, *p* < 0.001), whereas neither the main effect of treatment group (F = 0.486, *p* = 0.621) nor the treatment-group × visit interaction (F = 0.430, *p* = 0.656) was statistically significant. No statistically significant between-group differences were detected for PSS, PIS, or QOL at either Day 14 or Day 28 after Bonferroni adjustment. Overall, CBPI outcomes changed in a direction consistent with improvement during postoperative recovery, with no statistically significant treatment-group differences detected during the 28-day follow-up period.

#### 3.3.4. Radiographic Osteoarthritis

Radiographic osteoarthritis measures showed baseline differences among treatment groups for selected variables (Table 6). At Day 0, osteophytosis scores differed among treatment groups (Kruskal–Wallis *p* = 0.028), with higher scores in the ACS group than in the placebo group after Bonferroni adjustment (adjusted *p* = 0.037). Subjective OA scores also differed among groups at baseline (*p* = 0.018), with higher scores in both the ACS group (adjusted *p* = 0.040) and the ACS + CBD group (adjusted *p* = 0.046) than in the placebo group. The semiquantitative OA severity score also differed among groups at baseline (*p* = 0.016), with higher scores in the ACS + CBD group than in the placebo group (adjusted *p* = 0.025). No other baseline radiographic differences were statistically significant.

From baseline to postoperative Day 28, joint-effusion scores decreased within all treatment groups (ACS, *p* = 0.015; ACS + CBD, *p* = 0.006; placebo, *p* = 0.011). Subjective OA scores decreased within the ACS (*p* = 0.015) and ACS + CBD (*p* = 0.006) groups, whereas the within-group change in the placebo group was not statistically significant (*p* = 0.180). Osteophytosis scores increased within the placebo group (*p* = 0.025), whereas statistically significant within-group changes were not detected in the ACS or ACS + CBD groups. No statistically significant within-group changes were detected for intra-articular mineralization, tibial subchondral sclerosis, or semiquantitative OA severity scores.

On postoperative Day 28, no statistically significant between-group differences were detected for joint effusion, osteophytosis, intra-articular mineralization, tibial subchondral sclerosis, subjective OA score, or semiquantitative OA severity score (all *p* > 0.05). Given the baseline between-group differences and the exploratory nature of these ordinal outcomes, the within-group changes were interpreted descriptively as changes over time and were not considered evidence of comparative treatment effects.

#### 3.3.5. Radiographic Osteotomy Healing

Immediately after surgery, osteotomy-healing scores were 0 in all dogs, as expected before radiographic evidence of healing had developed. By postoperative Day 28, radiographic evidence of osteotomy healing was present in all treatment groups. Median 10-point TPLO healing scores were 3 (range, 1–5) in the placebo group, 3.5 (range, 1–7) in the ACS group, and 4 (range, 2–8) in the ACS + CBD group. Corresponding median 5-point healing scores were 2 (range, 1–3), 1.5 (range, 1–2), and 2 (range, 1–3), respectively (Table 7).

No statistically significant between-group differences were detected on postoperative Day 28 for cortical continuity (*p* = 0.719), osteotomy-line visibility (*p* = 0.287), callus formation/remodeling (*p* = 0.162), rounding of the distal osteotomy margin (*p* = 0.068), the 5-point healing scale (*p* = 0.311), or the 10-point TPLO healing score (*p* = 0.300). The ACS + CBD group had a numerically higher median 10-point TPLO healing score than the other groups, but the between-group comparison was not statistically significant.

Within-group increases in healing scores from the immediate postoperative assessment to Day 28 were consistent with expected postoperative healing progression and were interpreted descriptively rather than as evidence of comparative treatment effects.

### 3.4. Safety Outcomes

#### 3.4.1. Adverse Events and Postoperative Complications

One dog in the ACS group developed a surgical site infection that was detected on postoperative Day 3 and met a predefined withdrawal criterion. The dog was withdrawn from subsequent planned outcome assessments but remained in the safety population. Apart from this event, no additional clinically relevant adverse events or postoperative complications were observed during the 28-day study period. No rescue analgesia was required.

#### 3.4.2. Laboratory Safety Outcomes

Laboratory safety monitoring did not identify a consistent treatment-specific pattern of hematologic or serum biochemical abnormalities during the 28-day postoperative period (Supplementary Table S5). Longitudinal analyses of hematologic variables and renal- and protein-related biochemical measures showed no consistent statistically significant treatment-group differences. A statistically significant baseline difference in serum creatinine was identified, with higher concentrations in the ACS group than in the ACS + CBD group; however, no statistically significant between-group difference was identified on postoperative Day 28.

ALT activity did not differ significantly among treatment groups on postoperative Day 28. In contrast, ALP activity differed among groups on Day 28, with median activities of 32 U/L in the placebo group, 78 U/L in the ACS group, and 170.5 U/L in the ACS + CBD group. Bonferroni-adjusted pairwise comparisons showed higher ALP activity in the ACS + CBD group than in the placebo group (adjusted *p* = 0.011), whereas the ACS group did not differ significantly from either the placebo or ACS + CBD group. Using the laboratory reference interval of 1–114 U/L, the numbers of dogs with ALP activity above the upper reference limit on Day 28 were 1, 3, and 6 in the placebo, ACS, and ACS + CBD groups, respectively. This finding was not accompanied by a concurrent between-group difference in ALT activity. Apart from the Day 28 ALP finding, no broader consistent pattern of treatment-associated hematologic or biochemical abnormalities was identified during the 28-day follow-up period.

## 4. Discussion

The present exploratory randomized clinical trial evaluated early postoperative biomarker, clinical, biomechanical, owner-reported, radiographic, and safety outcomes in dogs receiving placebo, ACS, or ACS + CBD after TPLO. The main ARH(1) model showed a significant treatment-group effect, whereas the treatment-group × visit interaction did not reach statistical significance (*p* = 0.053). Exploratory visit-specific comparisons indicated lower CRP concentrations in the ACS + CBD group than in the placebo group on postoperative Days 3 and 5. Sensitivity analyses showed some variation according to model specification, particularly for the overall interaction and the Day 5 comparison. No consistent treatment-group pattern was identified across the clinical, biomechanical, owner-reported, or radiographic outcomes during the 28-day follow-up period. Accordingly, the CRP findings should be regarded as hypothesis-generating evidence of a possible early difference in systemic inflammatory activity rather than confirmatory evidence of treatment efficacy, and their clinical relevance remains uncertain.

The temporal CRP response observed in the present study was consistent with the expected systemic acute-phase response following orthopedic surgery [5,17,26]. Serum CRP concentrations increased markedly by postoperative Day 1 in all three groups and subsequently declined toward baseline. This pattern is consistent with previous canine orthopedic studies showing that CRP increases within hours after surgery, commonly reaches its maximum concentration approximately 12–24 h postoperatively, and subsequently declines during uncomplicated recovery [5,17]. The pronounced Day 1 increase observed across all three groups in the present study therefore most likely reflects the systemic inflammatory response to surgical trauma rather than a treatment-specific effect. Importantly, CRP is a sensitive but nonspecific marker of systemic inflammation and does not provide a direct measure of intra-articular inflammatory activity or clinical function [16,27].

Accordingly, the lower CRP concentrations observed in the ACS + CBD group than in the placebo group on postoperative Days 3 and 5 are consistent with a possible difference in the early postoperative systemic inflammatory response, but they do not establish treatment-mediated attenuation of inflammation or superior clinical recovery. The Day 3 difference was comparatively consistent across model specifications, whereas inference regarding the Day 5 comparison and the overall treatment-group × visit interaction was more sensitive to model specification. The absence of corresponding treatment-group differences in PVF, CBPI outcomes, or most orthopedic assessment domains further limits the interpretation of these early biomarker differences.

A biological basis for potential ACS-associated modulation of inflammatory signaling is plausible because ACS is generated by conditioning autologous blood to increase concentrations of anti-inflammatory mediators, particularly interleukin-1 receptor antagonist (IL-1Ra). IL-1Ra competitively inhibits interleukin-1 signaling, a pathway implicated in synovial inflammation, pain, and cartilage degradation in osteoarthritis [7]. In dogs, ACS has been shown to contain increased concentrations of IL-1Ra and selected growth factors relative to baseline plasma, with considerable interindividual variability in mediator concentrations [8]. Experimental work using ACS derived from dogs with osteoarthritis has further demonstrated anti-inflammatory and potentially regenerative cellular effects in vitro [9]. This variability suggests that administration of a fixed ACS volume may not represent an equivalent biological exposure across individuals. In the present study, IL-1Ra and other cytokine concentrations were not measured in individual ACS preparations; therefore, biological exposure could not be quantified or linked directly to the observed CRP differences.

The exploratory visit-specific CRP differences cannot be attributed specifically to either ACS or CBD. In the main ARH(1) model, CRP concentrations were lower in the ACS + CBD group than in the placebo group on postoperative Days 3 and 5. The ACS group did not differ significantly from placebo at these visits, and no significant differences were detected between the ACS and ACS + CBD groups. These non-significant comparisons should not be interpreted as evidence of equivalence or absence of an ACS effect.

From a biological perspective, both ACS and CBD have properties that could plausibly influence inflammatory signaling, although the present study cannot determine whether either mechanism contributed to the observed CRP pattern. As discussed above, ACS contains anti-inflammatory mediators capable of modulating IL-1-related signaling, whereas CBD has been reported to influence pain- and inflammation-related outcomes in dogs with osteoarthritis [10,11]. Cannabinoid receptors are also expressed on canine immune cells, providing a biological basis for potential immunomodulatory effects in dogs [14]. These observations provide biological context for the interventions evaluated but do not establish the mechanism responsible for the CRP differences in this study.

The present findings do not demonstrate that adding CBD to ACS provides additional benefit compared with ACS alone, nor do they establish that either component independently accounted for the observed biomarker finding. This interpretation is further limited by the absence of a CBD-only treatment group, which precluded direct assessment of the independent contribution of CBD. The three-group design therefore could not distinguish an ACS effect, a CBD-specific contribution, or a combined effect of the two interventions. Furthermore, systemic CBD exposure was not directly measured, limiting interpretation of any CBD-specific contribution.

The present findings should also be considered in relation to a recent randomized, double-blinded, placebo-controlled trial evaluating an oral 20:1 CBD:THC cannabis herbal extract in dogs undergoing TPLO [15]. In that study, administration of the extract at doses providing 2 or 5 mg/kg CBD twice daily for 14 days did not result in consistent improvements in postoperative pain or limb function compared with placebo, although transient differences in selected functional measures were observed at an early postoperative time point. Plasma cannabinoid concentrations were highly variable and were not associated with clinical outcomes [15]. Although the present study differed in formulation, treatment duration, and study design—including the use of CBD isolate at 2 mg/kg every 12 h in combination with intra-articular ACS—the absence of consistent treatment-group differences in PVF and owner-reported pain and function is broadly consistent with the limited evidence for an additional short-term clinical benefit of cannabinoid treatment during recovery after TPLO. The exploratory CRP finding in the present study cannot be directly compared with the findings of Lyons et al. because of differences in treatment formulation, co-intervention with ACS, outcome measures, and sampling schedules. Together, these studies indicate that the role of cannabinoids in acute postoperative recovery after TPLO remains uncertain and warrants further investigation using adequately powered trials with clearly prespecified clinical and biomarker endpoints.

The timing and frequency of intra-articular ACS administration could also influence treatment response, although an evidence-based dosing schedule has not been established in dogs. In the present study, ACS was administered intra-articularly immediately before skin closure on Day 0 and repeated on Day 14. Published canine reports have used different ACS administration strategies, including single or repeated injections, and no uniform repeat-injection interval has been established [28]. In equine practice, repeated ACS administration at approximately 2-week intervals has been used clinically, although this schedule has been based largely on clinical experience rather than comparative evidence [29]. Moreover, equine studies using different injection intervals have reported differences in synovial biomarker responses, without establishing the clinical superiority of any particular schedule. Therefore, the present study cannot determine whether a single perioperative injection, a more closely spaced early postoperative series, delayed repeat administration, or another dosing schedule would produce different biomarker or clinical responses. Future studies directly comparing alternative timing and repeat-administration strategies are needed to determine whether the ACS dosing schedule influences postoperative biomarker or clinical outcomes after TPLO.

Despite the early CRP differences observed in the ACS + CBD group, objective gait analysis did not demonstrate a corresponding treatment-group difference in operated-limb loading. Change from baseline in peak vertical force showed a statistically significant effect of visit, whereas neither the main effect of treatment group nor the treatment-group × visit interaction was statistically significant. The visit effect is consistent with increasing operated-limb loading during postoperative recovery after TPLO, while no statistically significant additional treatment-group differences were detected during the first 28 postoperative days. Previous studies have demonstrated progressive improvement in weight-bearing function during postoperative recovery after TPLO [3]. Accordingly, the relatively short follow-up period in the present study may have limited the ability to identify modest between-group differences in later biomechanical recovery. Although ΔPVF increased over time, no statistically significant between-group differences were detected at the postoperative time points evaluated. Because PVF was assessed on a different schedule from the early CRP measurements, the present data do not permit conclusions regarding the relationship between early postoperative CRP changes and subsequent objective limb loading.

Exploratory visit-specific differences were identified for the joint-mobility domain of the Orthopedic Assessment Score, although the treatment-group × visit interaction was not statistically significant. The ACS + CBD group showed a larger decrease in joint-mobility score from baseline than the placebo group on postoperative Days 7 and 28 and than the ACS group on Day 28. Previous clinical studies in dogs with osteoarthritis have reported potential beneficial effects of oral CBD on pain- and mobility-related outcomes, providing biological plausibility for a possible contribution of CBD to this observed pattern [10,11]. However, because no CBD-only group was included and corresponding treatment-group differences were not observed across PVF, CBPI, or the other OAS domains, the independent contribution of CBD cannot be determined. Moreover, baseline joint-mobility scores were numerically higher in the ACS + CBD group, indicating greater baseline impairment, although the between-group comparison did not reach statistical significance. Although the analyses were based on change from baseline, change scores may remain influenced by baseline values and regression toward the mean [30]. These considerations, together with the ordinal nature of the joint-mobility score and the non-significant treatment-group × visit interaction, limit interpretation of the visit-specific differences. Accordingly, the joint-mobility findings should be regarded as exploratory and hypothesis-generating rather than evidence of a consistent clinical benefit of ACS + CBD.

Owner-reported CBPI outcomes showed significant visit effects for the Pain Severity Score, Pain Interference Score, and quality-of-life score, whereas no significant main effects of treatment group or treatment-group × visit interactions were detected. No statistically significant between-group differences were identified at either Day 14 or Day 28, indicating that no differential treatment-group pattern was detected for owner-perceived pain severity, pain-related interference with function, or quality of life at these later postoperative assessments. However, CBPI was assessed only on Days 0, 14, and 28 and therefore did not coincide with the earlier postoperative interval during which between-group differences in CRP were observed on Days 3 and 5. By Days 14 and 28, the postoperative CRP response had largely resolved and no statistically significant between-group CRP differences were detected. Accordingly, the present data do not permit direct evaluation of the relationship between early postoperative CRP changes and owner-perceived pain or function. The CBPI findings therefore indicate that no statistically significant treatment-group differences in owner-reported outcomes were detected at the assessed postoperative time points. The standardized multimodal analgesic and rehabilitation protocols applied to all dogs may also have contributed to the overall postoperative improvement in CBPI scores and could have reduced the ability to identify modest additional between-group differences.

Radiographic osteoarthritis outcomes did not show evidence of a treatment-specific structural benefit during the 28-day follow-up period. Baseline differences were present for osteophytosis, subjective OA score, and semiquantitative OA severity score, with higher scores in one or both ACS-treated groups than in the placebo group. Although joint-effusion scores decreased within all treatment groups and subjective OA scores decreased within the ACS and ACS + CBD groups, no statistically significant between-group differences were detected for any radiographic OA outcome on postoperative Day 28. Given these baseline imbalances and the ordinal nature of the radiographic measures, the within-group changes should be interpreted descriptively rather than as evidence of comparative treatment effects. Previous studies have shown that radiographic OA may continue to progress for months to years after TPLO [6,31]. Accordingly, the 28-day follow-up period was too short to determine whether ACS or ACS + CBD affected longer-term structural progression of osteoarthritis.

Radiographic osteotomy healing progressed during the first 28 postoperative days in all treatment groups, with no statistically significant between-group differences in any individual healing component, the 5-point healing scale, or the 10-point TPLO healing score. Although the median 10-point TPLO healing score was numerically higher in the ACS + CBD group than in the other groups, the between-group difference was not statistically significant and should not be interpreted as evidence of accelerated bone healing. Conversely, no clear signal of impaired early radiographic osteotomy healing associated with ACS, either alone or combined with oral CBD, was identified under the conditions of the present study. Radiographic healing after TPLO continues beyond the first postoperative month, and previous studies have evaluated progressive osteotomy healing at approximately 6–8 weeks and later after surgery [32,33]. Accordingly, the Day 28 assessment reflects only an early stage of osteotomy healing and cannot determine whether the interventions affected later bone remodeling, time to radiographic union, or subsequent healing progression.

One dog in the ACS group developed a surgical site infection on postoperative Day 3 and was subsequently withdrawn from planned outcome assessments. This was the only clinically relevant adverse event observed during the study. Although a causal relationship between this event and ACS administration cannot be established, its occurrence should be considered when interpreting the short-term safety findings. Given the small sample size and limited follow-up, the present study cannot adequately assess treatment-related infection risk.

Short-term safety monitoring did not reveal a consistent treatment-specific pattern of hematologic or serum biochemical changes during the 28-day follow-up period, although an isolated between-group difference in ALP activity was identified on Day 28. ALP activity was statistically significantly higher in the ACS + CBD group than in the placebo group on Day 28, whereas ALT activity did not differ significantly among groups. Elevations in serum alkaline phosphatase activity have been reported in dogs receiving oral CBD, including in a clinical study using the same 2 mg/kg every 12 h dosing regimen as the present study and in controlled safety studies of repeated CBD administration [10,34]. The number of dogs with ALP activity above the upper reference limit was also numerically greater in the ACS + CBD group. However, because the present study did not include a CBD-only group and systemic CBD exposure was not measured, the observed ALP difference cannot be attributed specifically to CBD and should be regarded as a laboratory safety signal warranting further monitoring rather than evidence of a treatment-specific adverse effect.

Several limitations should be considered when interpreting the present findings. First, the small sample size limited statistical precision and the ability to detect modest between-group differences across the clinical and radiographic outcomes. In addition, the withdrawal of one dog in the ACS group following a surgical site infection resulted in missing CRP observations from Day 3 onward. Because the withdrawal was associated with a postoperative complication, the potential influence of these missing observations on the longitudinal CRP results cannot be excluded. Furthermore, inferential results for CRP showed some variation according to model specification, particularly for the treatment-group × visit interaction and the Day 5 comparison, underscoring the sensitivity of some findings to the analytical assumptions used in this small repeated-measures dataset. Second, the absence of a CBD-only treatment group precluded determination of the independent contribution of CBD and limited interpretation of the CRP differences observed in the ACS + CBD group. In addition, systemic CBD concentrations were not measured, and IL-1Ra or other cytokine concentrations were not quantified in individual ACS preparations; therefore, individual biological exposure to CBD or ACS mediators could not be quantified or related directly to biomarker or clinical responses. The study also evaluated only one ACS administration schedule, consisting of immediate postoperative injection and repeat injection on Day 14; therefore, the effects of alternative timing or repeat-administration schedules could not be assessed. Baseline imbalances were also present for selected radiographic OA variables, and joint-mobility scores were numerically higher in the ACS + CBD group at baseline, indicating greater baseline impairment and potentially influencing change-from-baseline estimates. Although post hoc baseline-adjusted and nonparametric sensitivity analyses were performed for joint mobility, these analyses do not eliminate the limitations associated with the small sample size and baseline imbalance. Finally, the 28-day follow-up period allowed characterization of early postoperative inflammatory and short-term functional responses but was too short to determine longer-term osteoarthritis progression, complete osteotomy healing, or sustained clinical benefit. The standardized multimodal analgesic and rehabilitation protocol provided consistent background care across groups but may have reduced the ability to identify modest incremental effects of the adjunctive interventions.

The effect estimates obtained in the present exploratory trial may help inform prospective sample-size planning for a future hypothesis-testing study. In the exploratory visit-specific analyses from the main ARH(1) model, CRP concentrations in the ACS + CBD group were approximately 48% and 44% lower than those in the placebo group on postoperative Days 3 and 5, respectively. Because these visit-specific estimates were obtained from an exploratory trial in which the overall treatment-group × visit interaction did not reach statistical significance in the main ARH(1) model, they may overestimate the true treatment effect and should not be used directly as the assumed effect size for a future confirmatory trial. For an illustrative planning scenario based on the Day 3 placebo response, a 40% between-group difference would correspond to an absolute difference of approximately 1.7 mg/dL. Assuming an SD of approximately 1.9–2.0 mg/dL, based on variance estimates from larger postoperative orthopedic cohorts and published TPLO data [17], a two-group trial comparing ACS + CBD with placebo would require approximately 22 evaluable dogs per group to achieve 80% power or 29 dogs per group to achieve 90% power at a two-sided alpha level of 0.05. These calculations are illustrative and are intended only to support prospective study planning. The target effect size and variance should be independently justified when designing a future trial, and allowance would also be required for anticipated attrition. These estimates should not be interpreted as a retrospective assessment of the statistical power or adequacy of the sample size of the present study.

Taken together, the present findings indicate that ACS combined with oral CBD was associated with lower serum CRP concentrations than placebo on postoperative Days 3 and 5 in exploratory visit-specific comparisons in the main ARH(1) model, although the treatment-group × visit interaction did not reach statistical significance in this model. Sensitivity analyses showed some variation according to model specification, particularly for the overall interaction and the Day 5 comparison. These differences are consistent with a possible transient difference in the early systemic inflammatory response following TPLO but do not establish treatment-mediated attenuation of inflammation. No concurrent between-group differences were detected in the OAS domains on postoperative Days 3 and 5, although exploratory differences in the joint-mobility domain were observed later on Days 7 and 28. Because PVF, CBPI, radiographic, and osteotomy-healing outcomes were assessed on different postoperative schedules, the present study was not designed to determine whether the early CRP differences were temporally related to subsequent clinical or structural outcomes.

The domain-specific joint-mobility findings should be interpreted cautiously. Although the original change-from-baseline analysis showed greater decreases in joint-mobility scores in the ACS + CBD group at selected postoperative visits, baseline scores were numerically higher in this group, indicating greater baseline impairment. Importantly, these visit-specific differences were not retained after adjustment for baseline joint mobility or in the nonparametric sensitivity analysis. Given the baseline numerical imbalance, the ordinal nature of the outcome, the non-significant treatment-group × visit interaction, and the lack of corresponding differences across the other OAS domains, the original joint-mobility findings should be regarded as exploratory and do not provide robust evidence of a treatment-related improvement in joint mobility. Overall, the current results do not establish a consistent short-term clinical advantage of ACS alone or ACS combined with CBD after TPLO. Rather, the findings provide preliminary effect estimates and hypotheses for evaluation in larger, prospectively designed studies with prespecified outcome and analysis strategies. A subsequent trial could prespecify the early CRP response as a biomarker endpoint while assessing clinical outcomes at corresponding postoperative time points to determine whether biomarker differences are associated with clinically meaningful recovery. A four-group design including placebo, ACS, CBD, and ACS + CBD would allow the independent and combined contributions of ACS and CBD to be evaluated directly. Future investigations should also evaluate alternative ACS dosing schedules and include longer-term functional and structural outcomes.

## 5. Conclusions

In this exploratory randomized clinical trial, no consistent treatment-group differences in short-term clinical or structural outcomes were identified during the first 28 days after TPLO. In the main ARH(1) model, dogs receiving ACS + CBD had lower serum CRP concentrations than dogs receiving placebo on postoperative Days 3 and 5 in exploratory visit-specific comparisons. Sensitivity analyses showed model-dependent variation in the overall treatment-group × visit interaction and the Day 5 comparison, whereas the Day 3 difference was comparatively consistent across the evaluated models. Given the exploratory multi-outcome design and the sensitivity of some CRP findings to model specification, these differences should be regarded as hypothesis-generating biomarker findings rather than confirmatory evidence of treatment efficacy. The clinical relevance of the early CRP differences remains uncertain because the study was not designed to determine whether they were associated with subsequent clinical or owner-reported recovery. Exploratory visit-specific differences were also identified in the joint-mobility domain in the original change-from-baseline analysis, but these differences were not retained after adjustment for baseline joint mobility or in the nonparametric sensitivity analysis. Accordingly, the joint-mobility findings should be interpreted cautiously given the baseline numerical imbalance, the ordinal nature of the outcome, and the absence of a consistent pattern across other clinical measures. Regarding short-term safety, one dog in the ACS group developed a surgical site infection on postoperative Day 3 and was withdrawn from subsequent outcome assessments. Higher ALP activity was also observed in the ACS + CBD group on Day 28. Larger, prospectively designed studies with appropriately timed biomarker and clinical assessments and longer follow-up are needed to determine whether ACS, CBD, or their combination provides clinically meaningful benefit after TPLO and to distinguish their independent and combined effects.

## Figures and Tables

**Table 1:** Orthopedic Assessment Scoring System Used for Clinical Evaluation of Dogs Undergoing TPLO.

Criterion	Clinical Evaluation
Lameness	1. Walks normally 2. Slightly lame when walking 3. Moderately lame when walking 4. Severely lame when walking 5. Reluctant to rise and will not walk more than five paces
Joint mobility	1. Full range of motion 2. Mild limitation (10–20%) in range of motion; no crepitus 3. Mild limitation (10–20%) in range of motion; with crepitus 4. Moderate limitation (20–50%) in range of motion; ± crepitus 5. Severe limitation (>50%) in range of motion; ± crepitus
Pain on palpation	1. None 2. Mild signs; dog turns head in recognition 3. Moderate signs; dog pulls limb away 4. Severe signs; dog vocalizes or becomes aggressive 5. Dog will not allow palpation
Weight-bearing	1. Equal on all limbs standing and walking 2. Normal standing; favors affected limb when walking 3. Partial weight-bearing standing and walking 4. Partial weight-bearing standing; non-weight-bearing walking 5. Non-weight-bearing standing and walking
Overall score of clinical condition	1. Not affected 2. Mildly affected 3. Moderately affected 4. Severely affected 5. Very severely affected

Note: Adapted from McCarthy et al. [21]. Each domain was scored from 1 to 5, with higher scores indicating greater clinical impairment.

**Table 2:** Baseline demographic and clinical characteristics of randomized dogs by treatment group.

Variable	Placebo (*n* = 9)	ACS (*n* = 9)	ACS + CBD (*n* = 9)	*p*-Value
**Continuous variables**				
Age, months	49.11 ± 31.03	64.56 ± 32.84	58.78 ± 29.83	0.578
Body weight, kg	33.11 ± 8.34	35.63 ± 8.45	38.13 ± 8.19	0.453
CRP, mg/dL	0.40 ± 0.32	0.42 ± 0.23	0.43 ± 0.31	0.970
PVF of operated pelvic limb, %BW	26.84 ± 14.15	21.36 ± 13.51	19.12 ± 18.23	0.584
OAS lameness score	2.78 ± 0.44	3.00 ± 0.50	3.22 ± 0.83	0.327
OAS joint mobility score	2.11 ± 0.78	2.44 ± 1.24	3.33 ± 1.00	0.055
OAS pain on palpation score	2.00 ± 1.00	2.00 ± 1.00	2.33 ± 1.12	0.738
OAS weight-bearing score	2.78 ± 0.44	3.11 ± 0.33	3.22 ± 0.67	0.168
OAS overall clinical condition score	2.89 ± 0.33	3.00 ± 0.50	3.11 ± 0.60	0.636
CBPI Pain Severity Score	16.89 ± 6.29	24.78 ± 10.85	23.78 ± 6.52	0.104
CBPI Pain Interference Score	32.89 ± 9.52	43.56 ± 14.54	43.44 ± 9.18	0.093
CBPI Quality-of-Life score	2.00 ± 0.71	1.89 ± 0.60	1.78 ± 0.67	0.777
**Categorical variables**				
**Sex, *n* (%)**				0.489
Male	5 (55.6)	4 (44.4)	2 (22.2)	
Female	4 (44.4)	5 (55.6)	7 (77.8)	
**Body condition score, *n* (%)**				0.357
4	2 (22.2)	0 (0.0)	0 (0.0)	
5	2 (22.2)	1 (11.1)	0 (0.0)	
6	2 (22.2)	1 (11.1)	2 (22.2)	
7	3 (33.3)	4 (44.4)	4 (44.4)	
8	0 (0.0)	3 (33.3)	3 (33.3)	
**Breed, *n* (%)**				0.944
American Bully	1 (11.1)	0 (0.0)	1 (11.1)	
American Pitbull	1 (11.1)	0 (0.0)	0 (0.0)	
Crossbreed	1 (11.1)	3 (33.3)	2 (22.2)	
Golden Retriever	0 (0.0)	1 (11.1)	1 (11.1)	
Labrador Retriever	2 (22.2)	1 (11.1)	2 (22.2)	
Rottweiler	2 (22.2)	1 (11.1)	0 (0.0)	
Siberian Husky	2 (22.2)	2 (22.2)	3 (33.3)	
Thai Bangkaew	0 (0.0)	1 (11.1)	0 (0.0)	
**Operated pelvic limb, *n* (%)**				0.386
Right	2 (22.2)	2 (22.2)	5 (55.6)	
Left	7 (77.8)	7 (77.8)	4 (44.4)	
**Medial meniscal tear, *n* (%)**				0.684
No	4 (44.4)	2 (22.2)	4 (44.4)	
Yes	5 (55.6)	7 (77.8)	5 (55.6)	

Note: Values are presented as mean ± SD for continuous variables and *n* (%) for categorical variables. *p*-values for continuous variables were obtained using one-way ANOVA, and *p*-values for categorical variables were obtained using Fisher’s exact test or its extension for larger contingency tables, as appropriate. ACS, autologous conditioned serum; CBD, cannabidiol; CRP, C-reactive protein; PVF, peak vertical force; %BW, percentage of body weight; OAS, orthopedic assessment score; CBPI, Canine Brief Pain Inventory. Bold text indicates variable categories and subgroup headings.

**Table 3:** Serum C-reactive protein (CRP) concentrations (mg/dL; estimated marginal means and 95% CIs) in dogs undergoing TPLO across the placebo, ACS, and ACS + CBD groups.

Postoperative Day	Placebo EMM (95% CI)	ACS EMM (95% CI)	ACS + CBD EMM (95% CI)	ACS vs. ACS + CBD Adjusted *p*-Value	ACS vs. Placebo Adjusted *p*-Value	ACS + CBD vs. Placebo Adjusted *p*-Value
Day 0	0.400 (0.189 to 0.611)	0.422 (0.212 to 0.633)	0.433 (0.223 to 0.644)	1.000	1.000	1.000
Day 1	6.322 (4.992 to 7.652)	5.089 (3.759 to 6.419)	4.622 (3.292 to 5.952)	1.000	0.569	0.224
Day 3	4.278 (3.401 to 5.155)	2.820 (1.897 to 3.742)	2.244 (1.367 to 3.122)	1.000	0.079	0.007
Day 5	2.000 (1.687 to 2.313)	1.617 (1.285 to 1.948)	1.122 (0.809 to 1.435)	0.103	0.289	0.001
Day 7	1.400 (1.012 to 1.788)	1.252 (0.840 to 1.664)	0.778 (0.389 to 1.166)	0.291	1.000	0.084
Day 14	1.389 (0.609 to 2.169)	0.670 (−0.163 to 1.503)	0.389 (−0.353 to 1.131)	1.000	0.608	0.200
Day 28	0.683 (0.415 to 0.951)	0.436 (0.130 to 0.742)	0.311 (0.045 to 0.577)	1.000	0.653	0.160

Note: Values are estimated marginal means (EMMs) with 95% confidence intervals (CIs) from the repeated-measures linear mixed model using a heterogeneous first-order autoregressive [ARH(1)] within-dog covariance structure. Pairwise treatment-group comparisons within each visit were adjusted using the Bonferroni method and were interpreted exploratorily because the overall treatment-group × visit interaction was not statistically significant (*p* = 0.053). The modified intention-to-treat (mITT) CRP analysis included all 27 randomized dogs. The dog withdrawn from the ACS group following detection of a surgical site infection on Day 3 contributed CRP measurements from Days 0 and 1 only; no subsequent CRP measurements were collected. ACS, autologous conditioned serum; CBD, cannabidiol; CI, confidence interval; EMM, estimated marginal mean; TPLO, tibial plateau leveling osteotomy.

**Table 4:** Change from baseline in peak vertical force (ΔPVF; %BW) of the operated pelvic limb across the placebo, ACS, and ACS + CBD groups.

Postoperative Day	Placebo EMM (95% CI)	ACS EMM (95% CI)	ACS + CBD EMM (95% CI)	ACS vs. ACS + CBD Adjusted *p*-Value	ACS vs. Placebo Adjusted *p*-Value	ACS + CBD vs. Placebo Adjusted *p*-Value
Day 7	−5.793 (−20.468 to 8.882)	5.200 (−10.365 to 20.766)	4.392 (−10.283 to 19.067)	1.000	0.896	0.962
Day 14	1.543 (−10.384 to 13.471)	6.796 (−5.855 to 19.447)	4.987 (−6.941 to 16.915)	1.000	1.000	1.000
Day 28	17.228 (6.377 to 28.079)	14.798 (2.900 to 26.696)	13.689 (2.838 to 24.539)	1.000	1.000	1.000

Note: Values are estimated marginal means (EMMs) with 95% confidence intervals (CIs) from the final repeated-measures linear mixed model of change from baseline using an unstructured within-dog covariance structure. Positive ΔPVF values indicate increased loading of the operated limb relative to Day 0. Pairwise treatment-group comparisons within each visit were adjusted using the Bonferroni method; no statistically significant between-group differences were identified at any postoperative visit. ACS, autologous conditioned serum; CBD, cannabidiol; CI, confidence interval; EMM, estimated marginal mean; PVF, peak vertical force; TPLO, tibial plateau leveling osteotomy; %BW, percentage of body weight.

**Table 5:** Change from baseline in the joint-mobility domain of the Orthopedic Assessment Score among treatment groups during the first 28 postoperative days.

Postoperative Day	Placebo EMM (95% CI)	ACS EMM (95% CI)	ACS + CBD EMM (95% CI)	ACS vs. ACS + CBD Adjusted *p*-Value	ACS vs. Placebo Adjusted *p*-Value	ACS + CBD vs. Placebo Adjusted *p*-Value
Day 1	1.333 (0.484 to 2.183)	1.375 (0.474 to 2.276)	0.444 (−0.405 to 1.294)	0.415	1.000	0.434
Day 3	0.889 (0.040 to 1.738)	0.625 (−0.276 to 1.526)	0.444 (−0.405 to 1.294)	1.000	1.000	1.000
Day 5	0.556 (−0.294 to 1.405)	0.875 (−0.026 to 1.776)	−0.222 (−1.071 to 0.627)	0.245	1.000	0.603
Day 7	1.000 (0.151 to 1.849)	0.000 (−0.901 to 0.901)	−0.667 (−1.516 to 0.183)	0.860	0.335	**0.022**
Day 14	0.081 (−0.790 to 0.952)	0.125 (−0.776 to 1.026)	−1.000 (−1.849 to −0.151)	0.223	1.000	0.242
Day 28	0.000 (−0.849 to 0.849)	−0.026 (−1.011 to 0.959)	−1.667 (−2.516 to −0.817)	**0.042**	1.000	**0.022**

Note: Values are estimated marginal means (EMMs) with 95% confidence intervals (CIs) from the repeated-measures linear mixed model of change from baseline. A first-order autoregressive [AR(1)] within-dog covariance structure was selected based on the lowest Akaike information criterion. Negative values indicate a decrease in joint-mobility score relative to baseline. Pairwise treatment-group comparisons within each visit were adjusted using the Bonferroni method. Bold values indicate statistically significant Bonferroni-adjusted pairwise comparisons (adjusted *p* < 0.05). The main effect of treatment group was statistically significant (F = 3.840, *p* = 0.033), whereas the treatment-group × visit interaction was not statistically significant (F = 1.457, *p* = 0.167). Baseline joint-mobility scores were numerically higher in the ACS + CBD group, indicating greater baseline impairment, although the baseline between-group comparison did not reach statistical significance (*p* = 0.055). In a post hoc baseline-adjusted sensitivity analysis, neither the treatment-group effect (*p* = 0.827) nor the treatment-group × visit interaction (*p* = 0.156) was statistically significant, and no between-group differences were detected at individual postoperative visits. A nonparametric sensitivity analysis of change-from-baseline scores likewise identified no significant between-group differences at any postoperative visit. ACS, autologous conditioned serum; CBD, cannabidiol; CI, confidence interval; EMM, estimated marginal mean; OAS, Orthopedic Assessment Score.

**Table 6:** Radiographic osteoarthritis scores at baseline and postoperative Day 28 among treatment groups.

Radiographic Outcome	Visit	Placebo Median (Range)	ACS Median (Range)	ACS + CBD Median (Range)	Kruskal–Wallis *p*-Value
Joint effusion	Day 0	2 (2–2)	2 (2–2)	2 (2–2)	1.000
	Day 28	1 (0–2)	0.5 (0–2)	1 (0–1)	0.202
Osteophytosis	Day 0	0 (0–1)	1 (0–1)	1 (0–2)	**0.028**
	Day 28	1 (0–2)	1 (0–1)	1 (0–2)	0.869
Intra-articular mineralization	Day 0	0 (0–0)	0 (0–1)	0 (0–1)	0.349
	Day 28	0 (0–0)	0 (0–1)	0 (0–1)	0.349
Tibial subchondral sclerosis	Day 0	1 (0–1)	1 (1–1)	1 (1–2)	0.112
	Day 28	1 (0–1)	1 (1–1)	1 (1–2)	0.112
Subjective OA score	Day 0	3 (2–4)	4 (3–5)	4 (3–6)	**0.018**
	Day 28	3 (1–4)	3 (1–4)	3 (1–5)	0.993
Objective OA severity score	Day 0	1 (1–2)	2 (2–3)	2 (1–4)	**0.016**
	Day 28	2 (1–3)	2 (2–3)	2 (1–4)	0.245

Note: Values are presented as median (range). Between-group comparisons at each visit were performed using the Kruskal–Wallis test. Where the overall between-group test was statistically significant, Bonferroni-adjusted pairwise comparisons were performed. Bold *p*-values indicate statistically significant overall between-group differences based on the Kruskal–Wallis test (*p* < 0.05), all of which occurred at baseline (Day 0). At baseline, statistically significant overall between-group differences were identified for osteophytosis (*p* = 0.028), subjective OA score (*p* = 0.018), and semiquantitative OA severity score (*p* = 0.016). No statistically significant between-group differences were identified for any radiographic OA outcome on postoperative Day 28. ACS, autologous conditioned serum; CBD, cannabidiol; OA, osteoarthritis.

**Table 7:** Radiographic osteotomy-healing scores on postoperative Day 28 in dogs undergoing TPLO across the placebo, ACS, and ACS + CBD groups.

Healing Outcome	Placebo (*n* = 9) Median (Range)	ACS (*n* = 8) Median (Range)	ACS + CBD (*n* = 9) Median (Range)	Kruskal–Wallis *p*-Value
Cortical continuity	1 (0–1)	1 (0–2)	0 (0–2)	0.719
Osteotomy-line visibility	1 (0–1)	1 (0–1)	1 (0–1)	0.287
Callus formation/remodeling	1 (1–2)	1.5 (1–2)	2 (1–3)	0.162
Rounding of distal osteotomy step	1 (0–1)	0.5 (0–2)	1 (1–2)	0.068
10-point TPLO healing score	3 (1–5)	3.5 (1–7)	4 (2–8)	0.300
5-point healing scale	2 (1–3)	1.5 (1–2)	2 (1–3)	0.311

Note: Values are presented as median (range). Immediate postoperative healing scores were 0 in all dogs, consistent with the absence of radiographic healing immediately after surgery. Higher scores indicate more advanced radiographic healing. Between-group comparisons at postoperative Day 28 were performed using the Kruskal–Wallis test. No statistically significant between-group differences were identified for any osteotomy-healing outcome on Day 28. ACS, autologous conditioned serum; CBD, cannabidiol; TPLO, tibial plateau leveling osteotomy.

## Data Availability

The data presented in this study are available within the article. Raw data supporting this study are available from the corresponding author upon reasonable request.

## Supplementary Materials

The following supporting information can be downloaded at: https://www.mdpi.com/article/10.3390/vetsci13101067/s1, Table S1: Subjective radiographic osteoarthritis (OA) scoring system; Table S2: Objective radiographic osteoarthritis (OA) severity scoring system (0–5); Table S3: Radiographic osteotomy healing assessment using a 10-point TPLO healing score; Table S4: Radiographic osteotomy healing assessment using a 5-point healing scale; Table S5: Longitudinal hematologic and serum biochemical findings in dogs treated with placebo, autologous conditioned serum (ACS), or ACS combined with cannabidiol (CBD) through postoperative Day 28. Table S6: Sensitivity analyses of longitudinal serum C-reactive protein (CRP) concentrations using alternative covariance structures and natural-log transformation.

## Abbreviations

The following abbreviations are used in this manuscript:

ACS	Autologous Conditioned Serum
ASA	American Society of Anesthesiologists
BCS	Body Condition Score
CBD	Cannabidiol
CBPI	Canine Brief Pain Inventory
CCLR	Cranial Cruciate Ligament Rupture
CRP	C-Reactive Protein
IL-1Ra	Interleukin-1 Receptor Antagonist
OA	Osteoarthritis
OAS	Orthopedic Assessment Score
PVF	Peak Vertical Force
QOL	Quality of Life
TPA	Tibial Plateau Angle
TPLO	Tibial Plateau Leveling Osteotomy
