# Supplementary material for: Intra-Articular Autologous Conditioned Serum Alone or Combined with Oral Cannabidiol in Dogs Undergoing Tibial Plateau Leveling Osteotomy: An Exploratory Randomized Controlled Trial
Source: Vet Sci. 2026 Oct 10;13(10):1067. doi: 10.3390/vetsci13101067 (PMC13709633; doi:10.3390/vetsci13101067)
Supplement: Supplementary file 1 [file vetsci-13-01067-s001.zip › vetsci-4565227-supplementary Table S1.pdf]

Supplementary Table S1. Subjective Radiographic Osteoarthritis (OA) Scoring System

Parameter	Score 0	Score 1	Score 2	Score 3
Joint effusion (0–2)	Absent	Moderate	Severe	—
Osteophytosis (0–3)	Absent	Slight	Moderate	Severe
Intra-articular mineralization (0–2)	Absent	Moderate	Severe	—
Tibial subchondral sclerosis (0–1)	Absent	Present	—	—

Note: The subjective OA scoring system was adapted from Innes JF et al. (2004).
