# Supplementary material for: Intra-Articular Autologous Conditioned Serum Alone or Combined with Oral Cannabidiol in Dogs Undergoing Tibial Plateau Leveling Osteotomy: An Exploratory Randomized Controlled Trial
Source: Vet Sci. 2026 Oct 10;13(10):1067. doi: 10.3390/vetsci13101067 (PMC13709633; doi:10.3390/vetsci13101067)
Supplement: Supplementary file 1 [file vetsci-13-01067-s001.zip › vetsci-4565227-supplementary Table S2.pdf]

Supplementary Table S2. Objective Radiographic Osteoarthritis (OA) Severity Scoring System (0–5)

Grade	Description	Radiographic Findings
0	Normal	No evidence of osteoarthritis (OA), joint effusion, or osteophytes
1	Early OA	Stifle joint effusion present; no osteophytes detected
2	Mild OA	Osteophytes present on the patella and femoral trochlear ridges only
3	Moderate OA	Small osteophytes on the patella, femoral trochlear ridges, femoral condyles, fabellae, periarticular margins of the tibial plateau, and fibular head
4	Moderate to severe OA	Medium to large osteophytes on the patella, femoral trochlear ridges, femoral condyles, fabellae, periarticular margins of the tibial plateau, and fibular head; mild to moderate subchondral sclerosis
5	Severe OA	Extensive osteophytes on the patella, femoral trochlear ridges, femoral condyles, fabellae, periarticular margins of the tibial plateau, fibular head, and intercondylar notch; marked calcification and subchondral sclerosis

Note: The objective OA severity scoring system was adapted from Moore EV et al. (2020).
