# Supplementary material for: Intra-Articular Autologous Conditioned Serum Alone or Combined with Oral Cannabidiol in Dogs Undergoing Tibial Plateau Leveling Osteotomy: An Exploratory Randomized Controlled Trial
Source: Vet Sci. 2026 Oct 10;13(10):1067. doi: 10.3390/vetsci13101067 (PMC13709633; doi:10.3390/vetsci13101067)
Supplement: Supplementary file 1 [file vetsci-13-01067-s001.zip › vetsci-4565227-supplementary Table S3.pdf]

Supplementary Table S3. Radiographic osteotomy healing assessment using a 10-point TPLO healing score

Component	Score	Definition
Cortical continuity	0	No cortical continuity
	1	One continuous cortex
	2	Two continuous cortices
Osteotomy line visibility	0	Osteotomy line clearly visible
	1	Osteotomy line barely visible
	2	Osteotomy line not visible
Callus formation / remodeling	0	None
	1	Minimal
	2	Moderate
	3	Remodeled
	4	Healed
Distal osteotomy step rounding	0	None
	1	Mild rounding
	2	Significant rounding

Note: The total healing score was calculated as the sum of all component scores and ranges from 0 to 10, where 0 indicates no radiographic evidence of healing and 10 indicates complete remodeling. The scoring system was adapted from Duerr FM et al. (2008), and Krotscheck U et al. (2012).
