# Supplementary material for: Intra-Articular Autologous Conditioned Serum Alone or Combined with Oral Cannabidiol in Dogs Undergoing Tibial Plateau Leveling Osteotomy: An Exploratory Randomized Controlled Trial
Source: Vet Sci. 2026 Oct 10;13(10):1067. doi: 10.3390/vetsci13101067 (PMC13709633; doi:10.3390/vetsci13101067)
Supplement: Supplementary file 1 [file vetsci-13-01067-s001.zip › vetsci-4565227-supplementary Table S4.pdf]

Supplementary Table S4. Radiographic osteotomy healing assessment using a 5-point healing scale

Score	Description
0	No radiographic evidence of healing (0% bridging)
1	Minimal healing (1–25% bridging)
2	Early healing (26–50% bridging)
3	Moderate healing (51–75% bridging)
4	Advanced healing (76–100% bridging)

Note: The 5-point healing scale (0–4) was used to estimate the percentage of radiographic bone bridging across the osteotomy site, where higher scores indicate more advanced healing. The scoring system was adapted from Duerr FM et al. (2008) and Krotscheck U et al. (2012).
