# Supplementary material for: Intra-Articular Autologous Conditioned Serum Alone or Combined with Oral Cannabidiol in Dogs Undergoing Tibial Plateau Leveling Osteotomy: An Exploratory Randomized Controlled Trial
Source: Vet Sci. 2026 Oct 10;13(10):1067. doi: 10.3390/vetsci13101067 (PMC13709633; doi:10.3390/vetsci13101067)
Supplement: Supplementary file 1 [file vetsci-13-01067-s001.zip › vetsci-4565227-supplementary Table S5.pdf]

Supplementary Table S5. Longitudinal hematologic and serum biochemical findings in dogs treated with placebo, autologous conditioned serum (ACS), or ACS combined with cannabidiol (CBD) through postoperative Day 28.

Parameter	Time	Placebo	ACS	ACS + CBD	Between-group p-value
PCV (%) Reference interval: 35–57 %	Day 0	42.9 (39.2–46.7)	40.9 (37.1–44.6)	42.0 (38.2–45.8)	0.734
	Day 3	39.7 (35.9-43.6)	40.3 (36.4-44.2)	40.7 (36.8-44.6)	0.934
	Day 14	42.7 (39.4-46.0)	41.1 (37.7-44.5)	42.2 (38.8-45.5)	0.788
	Day 28	42.6 (39.3–45.8)	42.7 (39.3–46.1)	44.2 (41.0–47.5)	0.711
WBC (cells/μL) Reference interval: 5,000–14,100 cells/μL	Day 0	12,308 (9,237–15,379)	13,701 (10,630–16,772)	12,282 (9,211–15,353)	0.745
	Day 3	13,068 (10,251-15,885)	16,143 (13,326-18,961)	12,362 (9,545-15,179)	0.136
	Day 14	13,670 (10,680-16,660)	14,975 (11,899-18,052)	12,960 (9,900-16,019)	0.629
	Day 28	13,081 (10,023–16,139)	13,157 (10,055–16,260)	12,404 (9,347–15,462)	0.925
Platelets (×10³/μL) Reference interval: 211-621 ×10³/μL	Day 0	307 (266–348)	299 (258–340)	248 (207–289)	0.095
	Day 3	283 (240-327)	279 (235-322)	225 (182-269)	0.123
	Day 14	361 (295-426)	305 (236-373)	292 (224-360)	0.294
	Day 28	395 (323–467)	301 (227–376)	274 (202–346)	0.054
BUN (mg/dL) Reference interval: 8-28 mg/dL	Day 0	17.56 (13.78–21.33)	12.78 (9.00–16.56)	15.44 (11.67–19.22)	0.202
	Day 3	11.78 (9.34-14.22)	9.22 (6.78-11.66)	10.44 (8.01-12.88)	0.327
	Day 14	13.33 (9.29-17.38)	14.16 (9.93-18.39)	11.91 (7.72-16.09)	0.732
	Day 28	13.78 (10.67–16.89)	13.89 (10.66–17.12)	13.67 (10.56–16.77)	0.995
Creatinine (mg/dL) Reference interval: 0.5-1.7 mg/dL	Day 0	1.04 (0.93–1.16)	1.21 (1.10–1.32)	0.97 (0.85–1.08)	**0.013**
	Day 3	0.91 (0.80-1.01)	1.09 (0.99-1.20)	0.90 (0.80-1.01)	**0.021**
	Day 14	0.97 (0.84-1.11)	1.10 (0.96-1.24)	0.95 (0.81-1.09)	0.272
	Day 28	1.02 (0.90–1.15)	1.19 (1.06–1.32)	0.98 (0.86–1.11)	0.061
ALT (U/L)† Reference interval: 10-109 U/L	Day 0	31 (15–63)	31 (20–283)	47 (27–276)	0.109
	Day 3	44 (25-80)	39 (23-91)	51 (27-141)	0.839
	Day 14	25 (18-75)	45.5 (18-215)	42 (21-105)	0.336
	Day 28	28 (19–48)	24 (21–156)	40 (15–74)	0.525
ALP (U/L)† Reference interval: 1-114 U/L	Day 0	43 (15–602)	76 (30–442)	89 (43–480)	0.219
	Day 3	68 (36-457)	79 (57-393)	91 (64-303)	0.354
	Day 14	43 (26-545)	83 (27-378)	74 (64-232)	0.162
	Day 28	32 (21–192)	78 (32–414)	170.5 (48–418)	**0.014**
Total protein (g/dL) Reference interval: 5.4-7.5 g/dL	Day 0	6.96 (6.48–7.43)	6.76 (6.28–7.23)	6.59 (6.11–7.07)	0.542
	Day 3	6.56 (6.15-7.00)	6.48 (6.07-6.89)	6.16 (5.75-6.56)	0.335
	Day 14	6.78 (6.37-7.20)	6.70 (6.27-7.13)	6.41 (5.99-6.84)	0.422
	Day 28	6.79 (6.45–7.12)	6.78 (6.44–7.13)	6.36 (6.02–6.69)	0.120
Albumin (g/dL) Reference interval: 2.3-3.1 g/dL	Day 0	3.26 (3.08–3.43)	3.19 (3.00–3.38)	3.21 (3.03–3.39)	0.864
	Day 3	3.14 (2.98-3.31)	3.06 (2.89-3.23)	3.02 (2.85-3.20)	0.562
	Day 14	3.20 (3.01-3.39)	3.11 (2.91-3.30)	3.20 (3.00-3.39)	0.742
	Day 28	3.19 (3.04–3.33)	3.22 (3.06–3.37)	3.12 (2.98–3.27)	0.641

Note: Values for PCV, WBC count, platelet count, BUN, creatinine, total protein, and albumin are presented as estimated marginal means (EMMs) with 95% confidence intervals derived from repeated-measures linear mixed models using an unstructured covariance structure.

† ALT and ALP values are presented as median (range) and were analyzed using nonparametric methods. For variables analyzed using linear mixed models, the between-group p-values shown at each visit represent the simple effect of treatment at that visit. Between-group p-values for ALT and ALP were obtained using the Kruskal–Wallis test at each visit.

All laboratory analyses included measurements obtained on Days 0, 3, 14, and 28. Bonferroni-adjusted pairwise comparisons showed higher creatinine concentrations in the ACS group than in the ACS + CBD group at baseline (adjusted $p = 0.013$) and on Day 3 (adjusted $p = 0.041$). On Day 28, ALP activity was higher in the ACS + CBD group than in the placebo group (adjusted $p = 0.011$). Using the laboratory reference interval of 1–114 U/L, the numbers of dogs with ALP activity above the upper reference limit on Day 28 were 1, 3, and 6 in the placebo, ACS, and ACS + CBD groups, respectively. This difference was not accompanied by a concurrent between-group difference in ALT activity.

All randomized dogs were included in the safety population, and all available laboratory observations were analyzed without imputation. ACS, autologous conditioned serum; ALP, alkaline phosphatase; ALT, alanine aminotransferase; BUN, blood urea nitrogen; CBD, cannabidiol; CI, confidence interval; EMM, estimated marginal mean; PCV, packed cell volume; WBC, white blood cell count.
