# Supplementary material for: Intra-Articular Autologous Conditioned Serum Alone or Combined with Oral Cannabidiol in Dogs Undergoing Tibial Plateau Leveling Osteotomy: An Exploratory Randomized Controlled Trial
Source: Vet Sci. 2026 Oct 10;13(10):1067. doi: 10.3390/vetsci13101067 (PMC13709633; doi:10.3390/vetsci13101067)
Supplement: Supplementary file 1 [file vetsci-13-01067-s001.zip › vetsci-4565227-supplementary Table S6.pdf]

Table S6. Sensitivity analyses of longitudinal serum C-reactive protein (CRP) concentrations using alternative covariance structures and natural-log transformation.

CRP scale	Covariance structure	AIC	Treatment group × visit p-value	ACS + CBD vs. placebo, Day 3 adjusted p-value	ACS + CBD vs. placebo, Day 5 adjusted p-value
Original scale	ARH(1)†	373.626	0.053	0.007	0.001
Original scale	CSH	374.674	0.029	0.013	0.002
Original scale	UN	379.131	0.066	0.018	0.004
Original scale	AR(1)	494.548	0.170	<0.001	0.231
Original scale	CS	496.390	0.107	<0.001	0.243
ln(CRP)	UN	264.660	0.375	0.016	0.010
ln(CRP)	ARH(1)	267.466	0.921	0.004	0.001
ln(CRP)	CSH	276.494	0.691	0.012	0.006

† ARH(1) was selected as the main covariance structure for CRP on the original scale based on the lowest AIC among the expanded candidate covariance structures. Visit-specific comparisons represent Bonferroni-adjusted pairwise comparisons and should be interpreted as exploratory. AIC values should be compared only among models fitted to the same response scale and should not be compared directly between original-scale CRP and ln(CRP) models.

Abbreviations: AIC, Akaike information criterion; AR(1), first-order autoregressive; ARH(1), heterogeneous first-order autoregressive; CS, compound symmetry; CSH, heterogeneous compound symmetry; UN, unstructured; ACS, autologous conditioned serum; CBD, cannabidiol.
